# PCFT‐Independent Cellular Uptake of Cyclic Cell‐Penetrating Peptide‐Conjugated Folic Acid

**DOI:** 10.1002/cbic.202500242

**Published:** 2025-05-30

**Authors:** Vineet Kumar Mishra, Rheal Towner, Juan Carlos Rodriguez‐Lecompte, Marya Ahmed

**Affiliations:** ^1^ Department of Chemistry Atlantic Veterinary College, University of Prince Edward Island 550 University Ave. Charlottetown PE C1A 4P3 Canada; ^2^ Faculty of Sustainable Design Engineering Atlantic Veterinary College, University of Prince Edward Island 550 University Ave. Charlottetown PE C1A 4P3 Canada; ^3^ Department of Pathology and Microbiology Atlantic Veterinary College, University of Prince Edward Island 550 University Ave. Charlottetown PE C1A 4P3 Canada

**Keywords:** dihydrofolate reductase assay, immunomodulation, peptide folic acid conjugates, proton‐coupled folate transporter downregulation

## Abstract

Folic acid is an essential component of many metabolic processes, including the synthesis of nucleoproteins, purines, and pyrimidines, and is a recommended supplement to lower the incidence of various disorders. Folic acid and folate‐loaded nanoparticles are extensively evaluated for sustained release and enhanced stability of the molecule; however, malfunctioning of proton‐coupled folate transporters (PCFTs) present on the intestinal cells and subsequent folate deficiency remain a major issue in this context. This study provides the first demonstration where cell‐penetrating peptide‐conjugated folic acid mediates PCFT‐independent folic acid permeabilization and intracellular bioavailability in vitro in the intestinal cells and macrophages. Cyclic‐transactivating transcriptional activator (cTAT) folic acid conjugates are prepared by solid‐phase peptide synthesis and are evaluated for the cellular uptake and bioavailability in the presence and absence of PCFT inhibitors. Compared with free folic acid that showed PCFT‐mediated cellular uptake, cTAT‐folic acid conjugates exhibited enhanced cellular uptake at all studied pH and improved intracellular bioavailability of the cargo, as was determined by dihydrofolate reductase (DHFR) assay. Folic acid and cTAT‐folic acid conjugates also dampened the production of proinflammatory mediators in the presence of toxins in vitro in macrophage cell lines.

## Graphical abstract

Cell‐penetrating peptide (CPP)‐conjugated folic acid mediates efficient internalization of folic acid that is independent of the presence of proton‐coupled folate transporter (PCFT) on the intestinal cells. CPP‐conjugated folic acid demonstrates superior cellular uptake at all studied pH and in the presence of PCFT inhibitors and shows enhanced bioavailability compared to free folic acid.

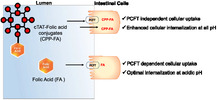

## 1 Introduction

Folate is an essential component of many metabolic processes, including the synthesis of nucleoproteins, purines, and pyrimidines mediated by one‐carbon (1C) transfer processes, and is a well‐documented molecule with immunomodulatory activities. Folate deficiency can cause several disorders, such as impaired DNA synthesis, reduced cell division, inflammation, and neural defects in embryos.^[^ ^1^, ^2^, ^3^, ^4^, ^5^, ^6^, ^7^, ^8^ ^]^ Folic acid, a protonated form of folate, is the most common supplement to overcome folate deficiency; however, malfunctioning of folate receptors due to acquired and hereditary disorders results in reduced uptake and bioavailability of folates in vitro and in vivo.^[^ ^9^, ^10^ ^]^ Among various folate receptors that are ubiquitously present throughout the organism, proton‐coupled folate transporter (PCFT) is one of the most studied ones, present at the apical brush border membrane of the intestinal cells and participate in pH‐ and concentration‐dependent uptake of folic acid/folates. Folate concentration dependent downregulation of PCFT and malfunctioning of the transporter under pathological conditions is known to cause poor adsorption of folic acid and folates across the intestine in vitro and in vivo.^[^ ^1^, ^10^ ^]^

The advances in nanotechnology are currently focused on the development of folic acid loaded physiologically stable and pH responsive nanoparticles for selective and slow release of the cargo at the intestinal pH and to improve the bioavailability of folic acid.^[^ ^1^, ^10^, ^11^, ^12^, ^13^ ^]^ Folic acid conjugated peptides, nanocarriers, and biopolymers are also developed to target and enhance the affinity and selectivity of the nanoformulations towards folate receptors.^[^ ^14^, ^15^, ^16^, ^17^ ^]^ However, folic acid‐based nanocarriers do not address the issue of folate receptor malfunctioning and are not typically studied for folate concentration dependent downregulation of the receptors in the gastrointestinal tract.^[^ ^1^, ^10^, ^18^, ^19^ ^]^ The development of folic acid conjugates that are not solely dependent on receptor ligand interactions and can cause cellular uptake of the cargo by membrane permeation, independent of the optimal functioning of folate receptors on the cell surface, may provide alternative approach to achieve folate uptake and metabolism in the intestinal cells.

Transactivating transcriptional activator (TAT) peptide, first derived from protein transduction domain of human immunodeficiency virus 1, is a highly cationic cell‐penetrating peptide (CPP), well documented for rapid translocation of proteins, small molecules, nucleic acid, and nanoparticles across biological membranes, in vitro and in vivo.^[^ ^20^, ^21^ ^]^ The recent advancements in peptide chemistry further indicate that peptide architecture and physicochemical properties of the cargo play key role in successful cell permeation and retention of biological activities of the conjugates. For example, cyclic‐TAT (cTAT) bioconjugates, prepared by cyclization of linear peptide, offer superior conformational stability and enhanced cellular uptake, resulting in improved transportation capabilities and physiological stability, of various biomolecules.^[^ ^21^, ^22^ ^]^ The bioconjugation of folic acid with anticancer peptide is shown to improve ligand selectivity towards folate receptors overexpressed on cancer cells;^[^ ^15^, ^16^, ^17^ ^]^ however, synthesis of folic acid conjugated CPPs is not yet explored to improve the cellular uptake and bioavailability of the molecule, independent of the expression of PCFT on the intestinal cells.

Given the superior cell permeating capabilities of cyclic peptides, we hypothesize that cTAT‐folic acid conjugates will demonstrate enhanced cellular uptake independent of PCFT status on the intestinal cells, providing a promising approach for folic acid enrichment intracellularly. Herein, for the first time, we explore the synthesis of folic acid conjugated cTAT peptides, evaluation of intracellular localization of the conjugates in the intestinal cells, in the presence and absence of folate receptor inhibitors and availability of folic acid conjugates intracellularly by dihydrofolate reductase assay (a pathway essential for the synthesis of purines, thymidylate, and amino acids).^[^ ^4^ ^]^ cTAT peptide is prepared by solid phase peptide synthesis and is conjugated with folic acid at the γ‐position of glutamic acid moiety by carbodiimide chemistry. cTAT‐folic acid conjugates are analyzed for the purity and molecular weight by reverse phase high performance liquid chromatography (RP‐HPLC) and electron spray ionization mass spectrometry (ESI‐MS), respectively. The fluorophore‐labeled folic acid and cTAT‐folic acid conjugates are compared for the cellular uptake in Caco‐2 cells in the presence and absence of PCFT inhibitors to evaluate the uptake and availability of the conjugates under impaired transporter conditions in vitro. The effect of folic acid and cTAT‐folic acid conjugates on PCFT expression is evaluated by Western blot (WB) analysis. cTAT‐folic acid conjugates are also evaluated for immunomodulatory potential of macrophages in vitro.

## 2 Experimental Section

### 2.1 Materials

Rink amide AM resin, Fmoc‐protected amino acids, and 2‐(1H‐benzotriazol‐1‐yl)‐1,1,3,3‐tetramethyluronium hexafluorophosphate (HBTU) were purchased from Matrix Innovation Inc. (Québec, QC, Canada), *N,N‐*Diisopropylethylamine (DIPEA), acetonitrile (ACN), trifluoroacetic acid (TFA), 0.25% trypsin‐EDTA Fluorescein maleimide, lipopolysaccharide (LPS), and other reagents were obtained from Sigma Aldrich (Oakville, ON, Canada) unless indicated otherwise. RAW 264.7, HD‐11, and CaCo‐2 were purchased from Cedarlane (Burlington, ON, Canada). 5(6)‐Carboxytetramethylrhodamine (TAMRA) was obtained from Fisher Scientific (Ottawa, ON, Canada). Folic acid‐negative Dulbecco's modified Eagle's medium (DMEM), complete DMEM, fetal bovine serum (FBS), and 0.25% trypsin‐EDTA were purchased from VWR Life Sciences (Mississauga, ON, Canada). CellTiter 96 AQueous Cell Proliferation Assay was purchased from Promega (Madison, WI, USA).

### 2.2 Peptide Synthesis and Purification

cTAT and its conjugates were synthesized by solid‐phase peptide synthesis (SPPS) using a Focus XC Peptide Synthesizer (AAPPTec) via Fmoc protection/deprotection chemistry, using rink amide resins, HBTU as an initiator, and DIPEA as a catalyst, according to previously reported methods.^[^ ^23^, ^24^ ^]^ The peptide sequences are provided in **Table** **1** and Table S1, Supporting Information.

**Table 1:** Key parameters of cTAT‐folic acid conjugates.

Sample	Sequence	Expected M.W [g mol^−1^]	Obtained M.W [g mol^−1^]	ACN elution [%]
Folic acid	Folic acid	441.4	441.4	34.06
cTAT‐folic acid	CONH_2_‐c[EYGRKKRRQRRRK]‐NH‐ Folic acid	2221.37	2221.14	34.3
cTAT‐folic acid‐FL	CONH_2_‐c[EYGRKKRRQRRRK]C(FL)‐NH‐folic acid	2834.36	2830.40	29.86
Folic acid‐FL	Folic acid‐C‐FL	1054.26	1058.70	22.7

*For peptide cyclisation:* Linear TAT peptide synthesized by SPPS on rink amide resins was modified with Alloc‐protected Lys and Glu at *N‐* and *C*‐ terminal of the peptide sequence, respectively. Alloc groups from Lys and Glu residues of linear peptide precursor were selectively removed using Pd(PPh_3_)_4_ (palladium and phenylsilane) (270 mg, 0.5 eq.) in CHCl_3_/AcOH/NMM (v/v ratio of 37/2/1), as a solvent, at room temperature for 2 h. The peptide containing resins were washed using 0.2 m DIPEA in DMF, and deprotection of Alloc groups was confirmed by ESI‐MS. Fifty milligrams of deprotected peptide containing resins were weighed in a 1 mL bed volume chromatography column and were washed with DCM, methanol, and DMF respectively, three times each. One hundred micromoles of HBTU, 200 μmol DIPEA, and 1 mL DMF were added to the resin, and the resin were incubated spinning for 2 h to achieve cyclization of peptides on resins.

*cTAT‐folic acid conjugates*: Cyclic peptide containing resins were washed with DCM, methanol, and DMF respectively, three times each. For the conjugation of folic acid, amino terminal of the peptide was deprotected by using 20% piperidine in 1 mL of DMF for 20 min, two times, and Fmoc deprotection was confirmed by Kaiser assay. One hundred micromoles of γ‐glutamic acid, 100 μmol HBTU, 200 μmol DIPEA, and 1 mL DMF were then added to the resin, and the resin was incubated spinning for 2 h. Glutamic acid‐modified resins were then modified with N^10^‐(trifluoroacetyl)pteroic acid using HBTU/DIPEA chemistry as indicated above. After the synthesis was complete, cTAT‐folic acid conjugates were cleaved in TFA:TIPS:H_2_O (95:25:25 v/v) mixture for 2.5 h at room temperature. The peptide containing cocktail was then poured in 10 mL diethyl ether and centrifuged at 2000 × g for 5 min. The supernatant was discarded, and the peptide was left to dry overnight.

Peptides were purified using a reverse‐phase high‐performance liquid chromatography (RP‐HPLC) system, Agilent Technologies (Santa Clara, CA, USA), with water and acetonitrile gradient (5%–95%), containing 0.1% TFA as the mobile phase. Pentafluorophenyl (PFP) column was used as the stationary phase, and samples were detected at the absorbance of 220 nm. Electronic spray ionization‐mass spectrometry (ESI‐MS) was used to determine the molecular weights of the synthesized peptides. Mass spectrometry data were provided by the AIMS Mass Spectrometry Laboratory at the University of Toronto.

*cTAT‐FA‐fluophore conjugates:* cTAT peptide containing resins were modified with cysteine (Cys) amino acid, followed by the addition of γ‐glutamic acid, and *N* ^10^‐(trifluoroacetyl)pteroic acid, respectively, as described above. Cys‐modified cTAT‐folic acid conjugates were cleaved and were reacted with fluorescein diacetate maleimide at 1:5 molar ratio in PBS, in the presence of tris(2‐carrboxyethyl)phosphine hydrochloride (TCEP) and EDTA for 2 h. Fluorescent‐cTAT‐folic acid (cTAT‐folic acid‐FL) were purified by RP‐HPLC and were confirmed by ESI‐MS for the molecular weight.

*Folic acid‐fluorophore conjugates:* Fluorescein conjugated folic acid was synthesized as a control. Briefly, 52.5 mg rink amide resin was Fmoc‐deprotected, followed by the addition of Cys, γ‐glutamic acid, and *N* ^10^‐(trifluoroacetyl)pteroic acid, respectively, using protection and deprotection method described above. Cys‐modified folic acid was cleaved and was reacted with fluorescein diacetate maleimide at 1:5 molar ratio in PBS, in the presence of tris (2‐carboxyethyl)phosphine hydrochloride (TCEP) and EDTA for 2 h. Fluorescent‐folic acid (folic acid‐FL) was purified by RP‐HPLC and was confirmed by ESI‐MS for the molecular weight.

### 2.3 Cytotoxicity Assay

HD‐11 chicken macrophages were cultured in DMEM containing 8% FBS and 2% chicken serum (CS). Caco‐2 cells were cultured in DMEM containing 20% FBS. Cells in growth phase (≈70% confluency) were incubated with 10 and 20 μm of cTAT and cTAT‐folic acid conjugates for 24 h. Cell viability was determined in triplicates by MTS assay using the CellTiter 96 AQueous Cell Proliferation Assay, as per the manufacturer's protocol.

### 2.4 Cellular Uptake Studies

Caco‐2 and HD‐11 cells were then seeded in six‐well plates at a density of 2.0 × 10^5^ cells per well, in 2 mL of RPMI no folic acid medium. After overnight incubation, the cells were further incubated for 4 h with fluorescent folic acid and with cTAT‐folic acid‐FL conjugates (4.5 μm) at 37 °C in a humidified atmosphere containing 5% CO_2_. The cells were washed with PBS, and relative fluorescence of the samples was analyzed with the microplate reader using excitation wavelength of 490 nm and emission wavelength of 520 nm.

### 2.5 WB Analysis

Caco‐2 cells were seeded in six well culture plate at 1 × 10^6^ cells per well in RPMI 1640 folic acid negative medium for 24 h followed by the addition of folic acid, cTAT, and cTAT‐folic acid conjugates (0.1–200 μm) for 4 h. 0.5 mL of pre‐chilled RIPA lysis buffer at 4 °C was added to Caco‐2 cells in six‐well plate. The cell samples were scraped and centrifuged at 14 000 × g to remove cellular debris. The amount of protein in cell lysate was measured by using BCA assay. The protein samples (25 μg) were loaded directly onto 12% Tris·HCl polyacrylamide gels using 6X SDS loading buffer (1:1) containing DTT, and gel was run for 1 h at 110 V. After SDS‐PAGE, proteins were transferred to nitrocellulose membrane for 80 min at 110 V (Bio‐rad, Hercules, California, USA). The protein containing nitrocellulose membranes were blocked with 10% skimmed milk in TBST (20 mm Tris, 135 mm NaCl, and 1% Tween 20, pH 7.6) for 45 min at room temperature, followed by the addition of primary antibody (anti‐PCFT antibody, Abcam) (dilution ratio 1:1000) overnight at 4 °C. The secondary antirabbit IgG‐horseradish peroxidase conjugate (Cell Signaling Technology; 1:5,000 in TBST) was then added to the membrane for 2 h at room temperature, and blots were developed with the Amersham ECL Plus reagent (GE Healthcare).

### 2.6 DHFR Assay

DHFR kit was purchased from Millipore Sigma and was used to analyze DHFR activity, according to manufacturer's protocol. DHFR enzyme catalyzes the reduction of dihydrofolic acid to tetrahydrofolic acid, which is reversible and NADPH‐dependent reaction.

Dihydrofolic acid + NADPH + H^+^ ↔ Tetrahydrofolic acid + NADP^+^

Briefly, 20 μm NADPH, 15 μm DHF, 5 μm DHFR, 50 mm Tris (pH 7.5), and 2 mg mL^−1^ of cell lysates from Caco‐2 samples treated with 4.5 μm of cTAT‐folic acid conjugates and 20 μm folic acid were added to obtain final volume of 100 μL. DHFR activity at room temperature was monitored by continuously measuring the drop in NADPH concentration, by taking the absorbance at 340 nm for up to 2.5 min.

### 2.7 IBDV Viral Titration

A modified live IBDV vaccine, UNIVAX‐BDmild strain (ST12) from MSD Animal Health, was reconstituted with10 mL of PBS, and 500 μL aliquots were stored at −80 °C, according to previously reported procedures.^[^ ^25^ ^]^ An aliquot of the vaccine was added to a tissue culture‐treated T‐25 flask containing confluent HD‐11 cells, and the flask was incubated at 37 °C with 5% CO_2_ for 1 h to reproduce the virus and to obtain multiplicity of infection (MOI = 1). Following this, 5 mL of media was added, and the cells were incubated for 72 h under the same conditions. After that, the supernatant was extracted, centrifuged for 10 min at 1000 × g, and 500 μL aliquots were taken out and kept at −80 °C to evaluate 50% tissue culture infective‐dose assay (TCID_50_) in HD‐11. For this purpose, six logarithmic serial dilutions of the virus supernatant described above were prepared, and 100 μL of each dilution was added to a 96‐well plate containing 2 × 10^4^ cells per well. The plate was incubated at 37 °C with 5% CO_2_ for 4 h, and TCID_50_ was evaluated by MTS assay, as described above.

### 2.8 Cytokine Release Studies

HD‐11 and RAW 264.7 cells were cultured in folic acid free RPMI containing 10% FBS and 1% penicillin–streptavidin solution. Cytokine release profile from HD‐11 chicken macrophage cells and RAW 264.7 human macrophage cells was studied by treating cells in growth phase (≈70% confluency) with 1 μg mL^−1^ LPS or with IBDV at TCID_50_ for 1 h, followed by the addition of folic acid (20 μm) and cTAT‐folic acid conjugates (4.5 μm) in serum‐free, folic acid‐negative RPMI‐1640 medium. After 24 h of incubation, cell culture supernatant were collected, and production of chicken IL‐1β and IFN‐β cytokines in HD‐11 was evaluated in triplicate by ELISA kits purchased from Genorise Scientific Inc. (Berwyn, PA 19 312, United States). The production of mouse IL‐1β, TNF‐α and IL‐10 was quantified in RAW 264.7 cells in triplicates by ELISA assay.

### 2.9 Statistical Analysis

Each experiment was performed in triplicates and was repeated at least two times for reproducibility. For statistical tests, sample size *n* = 3 was used for mean and standard deviation calculations for each statistical analysis. The statistical significance (p values) was obtained using the Student independent *t*‐test, using the following equation in origin pro software

(1) $$t=\frac{\left(x_{1}-x_{2}\right)}{\sqrt{\frac{{\left(s_{1}\right)}^{2}}{n_{1}}+\frac{{\left(s_{2}\right)}^{2}}{n_{2}}}}$$

where *s*_1_ and *s*_2_ represent standard deviations from two data sets, *n*_1_ and *n*_2_ represent the number of elements in each data set, and *x*_1_ and *x*_2_ represent the means of the two data sets.

## 3 Results

### 3.1 Synthesis and Characterization of Peptide Conjugates

**Scheme 1:**
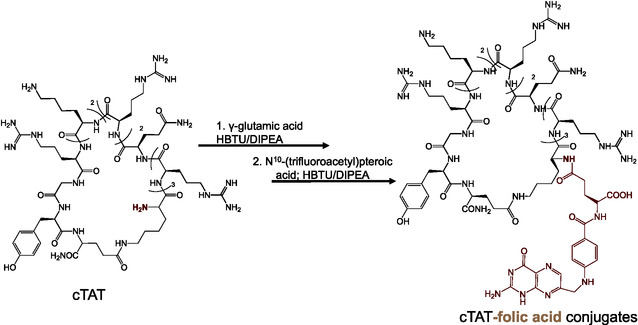
Schematics depicting the synthesis of cTAT‐folic acid conjugates by solid phase peptide synthesis, using Fmoc‐protection/deprotection chemistry.

The peptides and peptide‐folic acid conjugates were synthesized by solid phase peptide synthesis method, using Fmoc‐protection/deprotection chemistry. cTAT was synthesized from linear precursor by the macrocyclization of side‐chain to side‐chain by nonlabile amide bond formation (Figure S1–S2, Supporting Information). The peptides were modified with folic acid at the amino‐terminal of the cyclic CPP by HBTU/DIPEA chemistry, as shown in **Scheme** **1** (Figure S3, Supporting Information). Fluorescein‐labeled cTAT‐folic acid (cTAT‐folic acid‐FL) conjugates were prepared by incorporation of Cys amino acid between cTAT peptide sequence and γ‐glutamic acid residue of folic acid, followed by the conjugation of fluorescein at thiol residue of the peptide by maleimide chemistry (Figure S4, Supporting Information). The fluorophore‐labeled folic acid was also prepared on rink amide resins by incorporating cysteine as the first amino acid, followed by the addition of γ‐glutamic acid and *N* ^10^‐(trifluoroacetyl)pteroic acid. Cys‐modified folic acid and cTAT‐folic acid were cleaved and were coupled with the fluorophore by maleimide chemistry (Figure S5, Supporting Information).

The peptide conjugates were characterized by electrospray ionization mass spectrometry (ESI‐MS) with agreement between the predicted and experimental molecular weights (Table 1). The peptides, cTAT‐folic acid conjugates, and fluorescein‐labeled conjugates were assessed for the peptide purity by analytical RP‐HPLC system (Figure S6–S11, Supporting Information). The broader peaks, peak splitting, and lower signaling intensity of cyclic peptide and their conjugates in the chromatograms are due to the limited interactions of the peptides with the HPLC columns. The appearance of double peaks for TAMRA conjugated linear and cyclic peptides and of folic acid is likely due to the use of 5‐ and 6‐TAMRA isomer, since isomeric dye conjugated molecules tend to show multiple peaks in RP‐HPLC.^[^ ^23^ ^]^ Furthermore, peak intensity for cyclic peptides was partially improved by introducing trifluoroacetic acid (TFA) as a chaotropic agent. The bioconjugation of folic acid with cTAT increased the overall hydrophobicity of the cyclic peptide (%ACN elution change from 24% to 34% for cTAT and cTAT‐folic acid respectively), and the ACN elution profile of the conjugates was similar to that of folic acid (Table 1).

### 3.2 Physiological Stability and Cellular Uptake of Peptides

Compared with the linear peptides, cyclic analogs are well documented for enhanced physiological stability and resistance to enzymatic degradation.^[^ ^20^, ^21^, ^22^ ^]^ Linear and cyclic peptides subjected to trypsin treatment showed the presence of 36% and 49% intact peptide respectively, by HPLC chromatogram analysis post‐1 h incubation, whereas 15% and 27% intact peptides, respectively, were present 20 h post‐treatment, indicating higher physiological stability of the cyclic analog (Figure S12–S13, Table S1, Supporting Information). The evaluation of cellular uptake of by fluorescence microscopy and by quantifying the amount of fluorophore‐labeled peptides in cell lysates of HD‐11 and Caco‐2 cells by microplate reader demonstrated significantly higher cellular uptake of cyclic peptide compared with the linear analog. The cellular uptake of peptides was time dependent with superior uptake of cTAT within first 2 h of incubation, whereas linear peptide in general showed lower uptake at all studied time points (Figure S14–S15, Supporting Information); hence, further studies on folic acid conjugation were performed using cTAT peptide.

**Figure 1:**
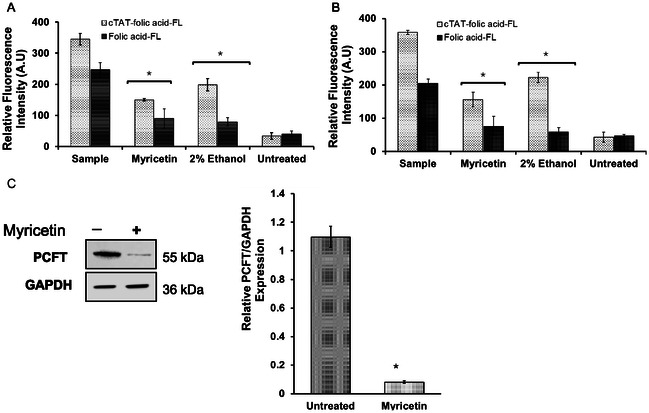
Cellular uptake of fluorescein‐labeled folic acid and cTAT‐folic acid conjugates (4.5 μm) in Caco‐2 cells in the presence and absence of inhibitors at A) acidic and B) physiological pH. C) WB analysis of PCFT/GAPDH expression in Caco‐2 cells in the presence of 50 μm myricetin. The experiments were replicated in triplicates (*n* = 3). The values are mean ± standard error for *n* = 3, *p < 0.01.

The treatment of intestinal cells and macrophages (Caco‐2, RAW264.7 and HD‐11 cells) with cTAT and cTAT‐folic acid conjugates demonstrated essentially nontoxic nature of the conjugates up to 10 μm concentration, whereas a slight decrease in cell viability was observed at higher concentrations (Figure S16, Supporting Information). The cellular uptake of fluorophore‐labeled folic acid (folic acid‐FL) and cTAT‐folic acid conjugates (cTAT‐folic acid‐FL) was studied at 4.5 μm concentration in Caco‐2 cells, at the physiological and acidic pH. The cellular uptake of folic acid‐FL in Caco‐2 cells was significantly higher at acidic pH, compared with the physiological pH and relative fluorescence intensity of the cells was 350 and 220 AU, respectively. However, no significant difference in cellular uptake of cTAT‐folic acid‐FL in Caco‐2 cells was observed at all studied pH. Furthermore, comparison of cellular uptake of folic acid with cTAT‐folic acid conjugates showed ≈1.5‐fold increase in cellular uptake of the conjugates, compared with folic acid at all studied pH (**Figure** **1**A,B).

The downregulation of PCFT at sublethal concentration of myricetin in Caco‐2 cells was evaluated by WB analysis (Figure 1C, Figure S17, Supporting Information), and PCFT‐mediated cellular uptake of folic acid‐FL and cTAT‐folic acid‐FL was compared in the presence and absence of myricetin. PCFT expression was significantly downregulated (*p* < 0.01) in Caco‐2 cells upon myricetin (50 μm) treatment, compared with the untreated cells. The cellular uptake of folic acid and folic acid conjugates in myricetin treated Caco‐2 cells also showed significantly reduced internalization of both folic acid‐FL and cTAT‐folic acid‐FL at the acidic and physiological pH, compared with the untreated cells (*p < 0.01). The cellular uptake of folic acid‐FL in the presence of myricetin was negligible and was comparable to that of untreated control, whereas ≈50% reduction in cellular uptake of cTAT‐folic acid‐FL conjugates was observed in the presence of myricetin.

The cellular uptake of Folic acid‐FL and cTAT‐folic acid‐FL upon ethanol treatment of Caco‐2 cells also showed significant reduction in uptake of Folic acid‐FL at both physiological and acidic pH, whereas cellular uptake of the cTAT‐folic acid conjugates was only partially inhibited (Figure 1A,B).

### 3.3 Intracellular Availability of cTAT‐Folic Acid Conjugates

#### 3.3.1 PCFT Expression Analysis

**Figure 2:**
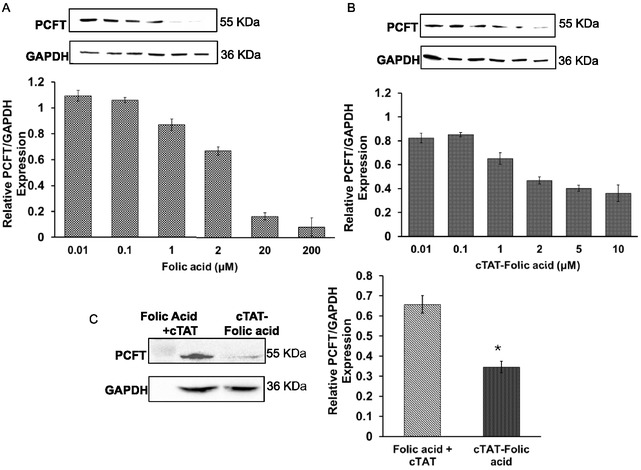
PCFT/GAPDH expression in Caco‐2 cells was measured by the WBs as a function of concentration of, A) folic acid, B) cTAT‐folic acid conjugates, and C) comparison of cTAT‐folic acid conjugates (4.5 μm) with unconjugated cTAT + folic acid (4.5 μm).The experiments were replicated twice in triplicates (*n* = 3) and upon 4 h post‐treatment. The values are mean ± standard error for *n* = 3, *p < 0.01.

The changes in PCFT expression as a function of folic acid concentration was evaluated by WB analysis (**Figure** **2**). Consistent with previous studies,^[^ ^1^, ^5^, ^10^ ^]^ the folic acid concentration (0.01–200 μm)‐dependent downregulation of PCFT in Caco‐2 cells was observed, with significant downregulation of the transporter (≈80% compared to untreated control) upon treatment with 20 μm folic acid (Figure 2a).

The concentration‐dependent PCFT downregulation was also observed in Caco‐2 cells treated with cTAT‐folic acid conjugates, and ≈50% PCFT downregulation was achieved at 2 μm concentration (Figure 2B). Further increase in cTAT‐folic acid conjugates concentration decreased PCFT expression, with ≈70% downregulation achieved at 10 μm concentration. WB analysis of PCFT downregulation in Caco‐2 cells above 10 μm concentration was not performed, due to the slight toxicity of the conjugates at the higher concentrations (Figure S16, Supporting Information). The comparison of PCFT downregulation in Caco‐2 cells upon treatment with equimolar concentration of unconjugated folic acid and cTAT (cTAT + FA) and cTAT‐folic acid conjugates (cTAT‐FA) showed ≈30%–40% PCFT downregulation by the unconjugated mixture that was comparable to that of folic acid alone. cTAT‐folic acid conjugates, however, showed superior PCFT downregulation efficacies (70%) suggesting higher bioavailability of the conjugates at the reduced concentration (4.5 μm). Furthermore, reduced PCFT expression in Caco‐2 cells upon treatment with cTAT‐folic acid conjugates, compared to unconjugated folic acid and cTAT, indicates that presence of free CPP is insufficient to enhance the bioavailability of folic acid by other processes such as membrane permeabilization (Figure 2C).

#### 3.3.2 DHFR Assay

**Figure 3:**
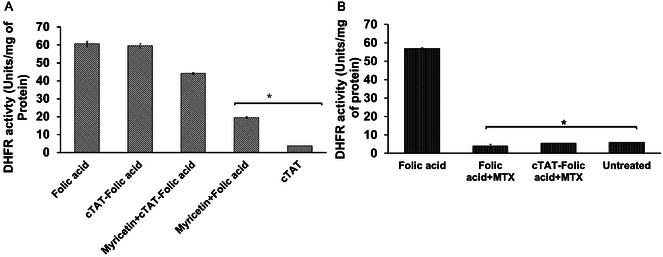
DHFR activity in units/mg of protein in Caco‐2 cells lysates, performed 4‐h post‐treatment. A) Cotreatment of cells with folic acid, cTAT‐folic acid conjugates, and myricetin and B) treatment of cell lysates with methotrexate (MTX). The values are mean ± standard error for *n* = 3, *p < 0.01. Units per mg indicate DHFR activity per mg of protein in the sample, where total protein content (mg) in each sample was measured by BCA assay.

Folic acid and cTAT‐folic acid conjugates treated Caco‐2 cells at the acidic pH were tested for DHFR activity in the cell lysates. Folic acid (20 μm) and cTAT‐folic acid conjugate (4.5 μm)‐treated Caco‐2 cell lysates showed high DHFR activity (59 and 61 units, respectively), indicating bioavailability of the conjugates post‐cellular internalization (**Figure** **3**A). cTAT peptide alone showed no significant DHFR activity, and values were comparable to that of untreated control. Consistent with the cellular uptake data, cotreatment with myricetin and folic acid significantly reduced DHFR activity (from 59 to 10 units mg^−1^ of protein) in Caco‐2 cell lysates, possibly due to PCFT expression inhibition and PCFT‐mediated cellular uptake of folic acid. The cotreatment of Caco‐2 cells with cTAT‐folic acid conjugates and myricetin resulted in insignificant reduction of DHFR activity (61 versus 55 units mg^−1^ of protein) in cell lysates. Combined with cellular uptake data in Figure 1, our results indicate that suppression of PCFT only partially inhibits cellular uptake of cTAT‐folic acid, and lower concentrations of the conjugates (4.5 μm of conjugates versus 20 μm of folic acid) are sufficient to participate in downstream folic acid pathways intracellularly. Methotrexate (MTX; 100 μm), a potent inhibitor of DHFR, is typically incorporated in cell lysates to evaluate the background activity of the protein in cell samples.^[^ ^26^ ^]^ MTX‐treated cell lysates showed negligible DHFR activity in folic acid and cTAT‐folic acid samples, suggesting DHFR activity is mainly mediated by ligand receptor interactions for both folic acid and cTAT‐folic acid conjugates treated samples.

#### 3.3.3 Immunomodulatory Activity of cTAT‐Folic Acid Conjugates

**Figure 4:**
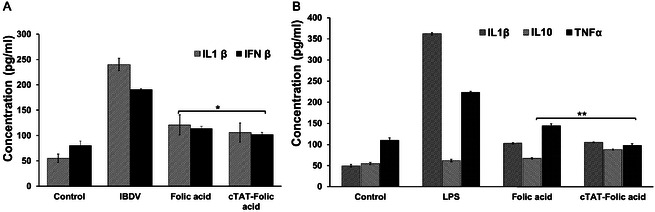
ELISA assay showing A) cytokine expression in HD‐11 cells upon IBDV infection for 1 h, followed by the treatment with folic acid (20 μm) and cTAT‐folic acid conjugates (4.5 μm), and B) cytokine expression (IL‐1β, IL‐10, and TNF‐α) upon LPS (0.5 μg mL^−1^) challenge of RAW 264.7 for 1 h, followed by the treatment with folic acid (20 μm) and cTAT‐folic acid conjugates (4.5 μm) for 4 h. The values are mean ± standard error for *n* = 3, *p < 0.01 and **p < 0.05.

The suppression of proinflammatory mediators upon acute LPS and infectious bursal disease virus (IBDV) challenge in macrophages was evaluated by the changes in production of cytokines (IL‐1β, IFNβ, TNF‐α, and IL‐10) in vitro (**Figure** **4**A,B, Figure S18, Supporting Information). As expected, LPS and IBDV treatment at tissue culture infectious dose 50 (TCID_50_)) significantly upregulated the production of proinflammatory cytokines (TNF‐α, IL‐1β, and IFNβ) in macrophages (RAW264.7, HD‐11). Folic acid (20 μm) and cTAT‐folic acid conjugate (4.5 μm) treatment of challenged macrophages, 1 h post‐infection, overall reduced the production of proinflammatory cytokines (*p < 0.01). Comparable reduction in proinflammatory mediator production in challenged macrophages, upon treatment with folic acid and cTAT‐folic acid conjugates, suggests superior immunomodulatory potential of the peptide conjugates at lower concentrations.

## 4 Discussion

Folic acid‐conjugated cyclic TAT peptide and fluorophore analogs were prepared upon sidechain to sidechain cyclization of linear TAT peptide, followed by the addition of fluorophore by maleimide chemistry. The site‐specific modification of amino terminal of the peptide with γ‐carboxylate of glutamic acid, followed by amidation with *N* ^10^‐(trifluoroacetyl)pteroic acid yielded cTAT‐folic acid conjugates (Scheme 1) The selective conjugation of folic acid at the γ‐carboxylic acid is key for the bioavailability of the cargo and for the recognition of conjugates by the folate receptors.^[^ ^14^, ^24^ ^]^ The cyclic peptide and corresponding conjugates showed broader peaks, peak splitting, and lower signal intensity in the chromatograms, possibly due to limiting interactions of the cyclic peptides with HPLC columns.^[^ ^23^ ^]^ The presence of double peaks in TAMRA‐labeled peptide chromatograms were attributed to the presence of TAMRA isomers that appear as two peaks in HPLC. The relative changes in overall hydrophobicity of the peptides post‐folic acid conjugation were comparable to the folic acid alone (Table 1), suggesting that the cargo maintains its physicochemical properties post‐conjugation with the peptide. Folic acid/folate adsorption in the intestinal epithelial cells is a pH‐dependent process and is mediated by a variety of folate receptors present on the cells surface at the physiological pH.^[^ ^25^, ^27^, ^28^ ^]^ PCFT in particular works optimally at the acidic pH and is mainly responsible for folic acid uptake, where contribution of other folate receptors is minimal.^[^ ^12^, ^28^ ^]^ Consistent with previous reports, folic acid alone demonstrated superior cellular uptake at the acidic pH, whereas cellular uptake was inhibited by myricetin, a flavonoid that binds and downregulates PCFT, hence reducing the intestinal folate transport in Caco‐2 cells. The fluorescently labeled cTAT‐folic acid conjugates, however, showed superior cellular uptake at all studied pH, and cellular uptake of the conjugates was only partially inhibited in the presence of PCFT inhibitor (Figure 1), possibly due to transporter independent interactions and internalization of the conjugates by membrane permeabilization in Caco‐2 cells. However, the exact mechanism of cellular uptake of folic acid peptide conjugates in Caco‐2 cells was not investigated.

Similarly, ethanol is a well‐documented for folate malabsorption and is shown to alter methylation pattern of folate receptor genes, causing PCFT downregulation.^[^ ^18^ ^]^ Consistent with previous studies, ethanol treatment significantly reduced cellular uptake of folic acid; however, uptake of the conjugates was only slightly reduced. Comparison of cellular uptake of folic acid with cTAT‐folic acid conjugates in the presence of inhibitors (myricetin and ethanol) reveals that folic acid uptake is mainly driven by PCFT in Caco‐2 cells and inhibition of PCFT drastically reduces cellular uptake of folic acid. In contrast, cTAT‐folic acid conjugates uptake is only partially mediated by PCFT and other mechanisms such as cell permeation may be responsible for enhanced intracellular delivery of the conjugates, independent of the expression and functioning of PCFT.

PCFT expression in intestinal cells is regulated by the folic acid concentration and oversupplementation of folic acid downregulates the transporter by the possible involvement of post‐transcriptional and translational regulatory mechanisms during intestinal folate regulation.^[^ ^27^, ^28^ ^]^ Interestingly, both folic acid and cTAT‐folic acid conjugates showed concentration dependent PCFT downregulation (Figure 2). The presence of unconjugated cTAT and folic acid in a treatment did not mediate PCFT downregulation, suggesting that bioconjugation of CPP with the cargo is critical for the membrane permeabilization and intracellular bioavailability of the cargo. The intracellular bioavailability of the conjugates was further confirmed by DHFR assay. DHFR catalyzes the reduction of dihydrofolate (DHF) to tetrahydrofolate (THF) in the presence of NADPH, and this folate pathway is essential for the synthesis of purines, thymidylate, and amino acids. DHFR assay is commonly used to evaluate folate pathway inhibitors and to study the bioavailability of folic acid treatments in vitro and in vivo.^[^ ^26^, ^29^ ^]^ Consistent with cellular uptake analysis, DHFR activity of folic acid treated samples was significantly reduced in the presence of inhibitors (myricetin and upon ethanol treatment), whereas DHFR activity remained essentially unchanged in the samples treated with cTAT‐folic acid conjugates. The cellular uptake analysis combined with DHFR activity suggest higher cellular uptake and bioavailability of cTAT‐folic acid conjugates compared with folic acid that is not solely dependent on the function of PCFT in the intestine cells.

Folic acid/folate supplementation is well documented to reduce chronic inflammation in immune cells (macrophages, monocytes, and microglia) possibly by downregulating mitogen‐activated protein kinases (MAPK) and nuclear factor‐kappa B (NF‐**κ**B) pathways, hence inhibiting the production of proinflammatory cytokines in vitro and in clinical settings.^[^ ^30^, ^31^, ^32^, ^33^ ^]^ Folic acid and cTAT‐folic acid conjugates effectively dampened the production of proinflammatory mediators released upon the infection of macrophages with LPS and IBDV; however, lower concentrations of the conjugates were required to achieve similar therapeutic effect.

## 5 Conclusions and Future Directions

cTAT‐folic acid conjugates were prepared by solid phase peptide synthesis by the site‐specific conjugation of glutamic acid moiety of folic acid with amino terminal of the cyclic peptide by carbodiimide chemistry. The fluorophore‐labeled folic acid and cTAT‐folic acid conjugates showed PCFT dependent cellular uptake of folic acid in Caco‐2 cells that was completely inhibited upon PCFT downregulation. The cellular uptake of cTAT‐folic acid‐FL was not only significantly higher than the folic acid but was also partially affected by the presence of PCFT inhibitor, suggesting PCFT independent cellular uptake of the conjugates by membrane permeabilization. The evaluation of PCFT downregulation as a function of folic acid concentration by WB analysis showed significant downregulation (>80%) of the receptor at high concentrations (20 μm). cTAT‐folic acid conjugates also showed concentration‐dependent PCFT downregulation with >50% downregulation achieved at lower concentrations (>2μm).

The intracellular bioavailability of the conjugates evaluated by DHFR assay further confirmed superior bioavailability of the conjugates at lower concentration (4.5 μm) compared with the folic acid (20 μm). PCFT inhibition by myricetin completely abrogated DHFR activity of folic acid; however, there was no significant reduction in DHFR activity for cTAT‐folic acid conjugate‐treated cell lysates. The reduced cellular uptake of folic acid, and complete inhibition of DHFR activity in the presence of myricetin, confirms PCFT‐mediated cellular uptake of folic acid. In contrast, superior cellular uptake of cTAT‐folic acid in the presence and absence of inhibitors and negligible effect on DHFR activity upon PCFT downregulation strongly suggest PCFT‐independent cellular uptake and higher bioavailability of the conjugates. Furthermore, folic acid and cTAT‐folic acid conjugates showed comparable immunomodulatory potential and reduced the production of proinflammatory mediators in macrophages challenged with toxins and upon viral infection. Future work will focus on evaluation of in vivo bioavailability of cTAT‐folic acid conjugates in mouse intestine.

## Conflict of Interest

The authors declare no conflict of interest.

## Author Contributions

**Vineet Kumar Mishra**: data curation (lead); formal analysis (lead); methodology (lead); writing—original draft (supporting). **Rheal Towner**: supervision (supporting); writing—review and editing (supporting). **Juan Carlos Rodriguez‐Lecompte**: funding acquisition (supporting); methodology (supporting); resources (supporting); writing—review and editing (supporting). **Marya Ahmed**: (lead); resources (lead); supervision (lead); writing—review and editing (lead).

## Supporting information

Supplementary Material

## Data Availability

The data that support the findings of this study are available in the supplementary material of this article.
