# Supplementary material for: PCFT‐Independent Cellular Uptake of Cyclic Cell‐Penetrating Peptide‐Conjugated Folic Acid
Source: Chembiochem. 2025 May 30;26(13):e202500242. doi: 10.1002/cbic.202500242 (PMC12247024; doi:10.1002/cbic.202500242)
Supplement: Supplementary file 1 — Supplementary Material [file CBIC-26-e202500242-s001.pdf]

Research article

Title Evaluating Bis-phenacyl bromide-based Bis-heterocyclic templates as anticancer prototypes and potential PARP1 inhibitors

Authors' names Refaie M. Kassab,[a]
Mohamed A. M. Ali,* [b]
Sami A. Al-Hussain,[c]
Magdi E. A. Zaki,[c]
Zeinab A. Muhammad,[d] and
Alyaa S. Abdel Halim[e]

Authors' affiliations [a] Department of Chemistry, Faculty of Science, Cairo University, Giza 12613, Egypt;
[b] Department of Biology, College of Science, Imam Mohammad Ibn Saud Islamic University (IMSIU), Riyadh 11623, Saudi Arabia;
[c] Department of Chemistry, College of Science, Imam Mohammad Ibn Saud Islamic University (IMSIU), Riyadh 11623, Saudi Arabia;
[d] Pharmaceutical Chemistry Department, Egyptian Drug Authority (EDA), Giza 12311, Egypt;
[e] Department of Biochemistry, Faculty of Science, Ain Shams University, Cairo 11566, Egypt

Correspondence Mohamed A. M. Ali, [b]Department of Biology, College of Science, Imam Mohammad Ibn Saud Islamic University (IMSIU), Riyadh 11623, Saudi Arabia; mamzaid@imamu.edu.sa

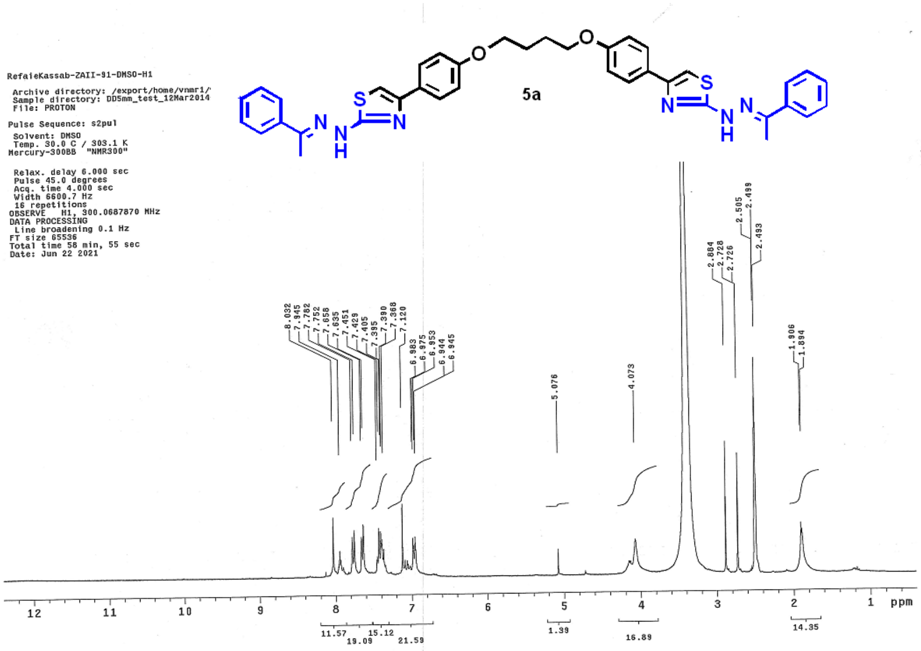

^{1}H NMR spectrum compound 5a

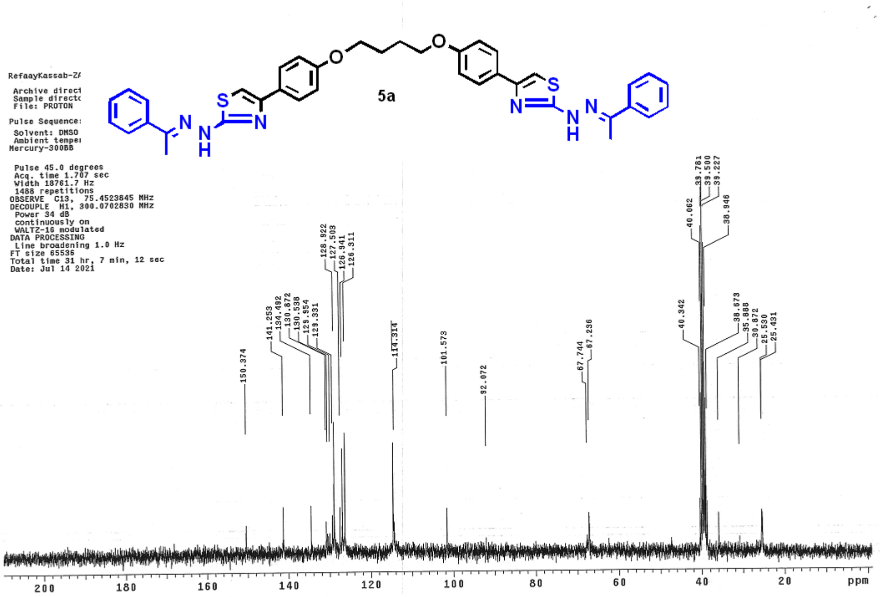

^{13}C NMR spectrum compound 5a

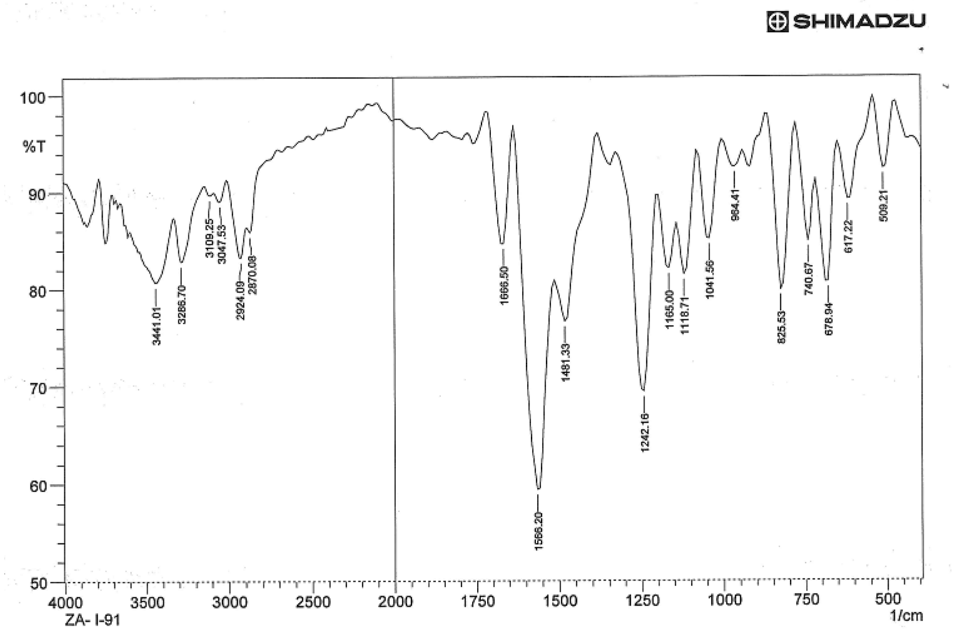

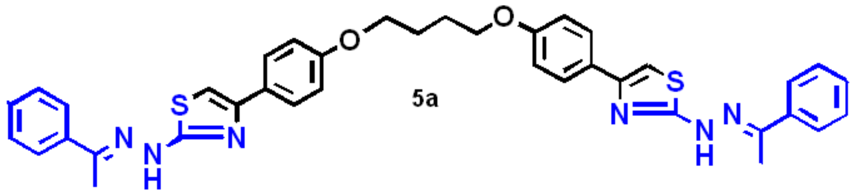

IR spectrum compound 5a

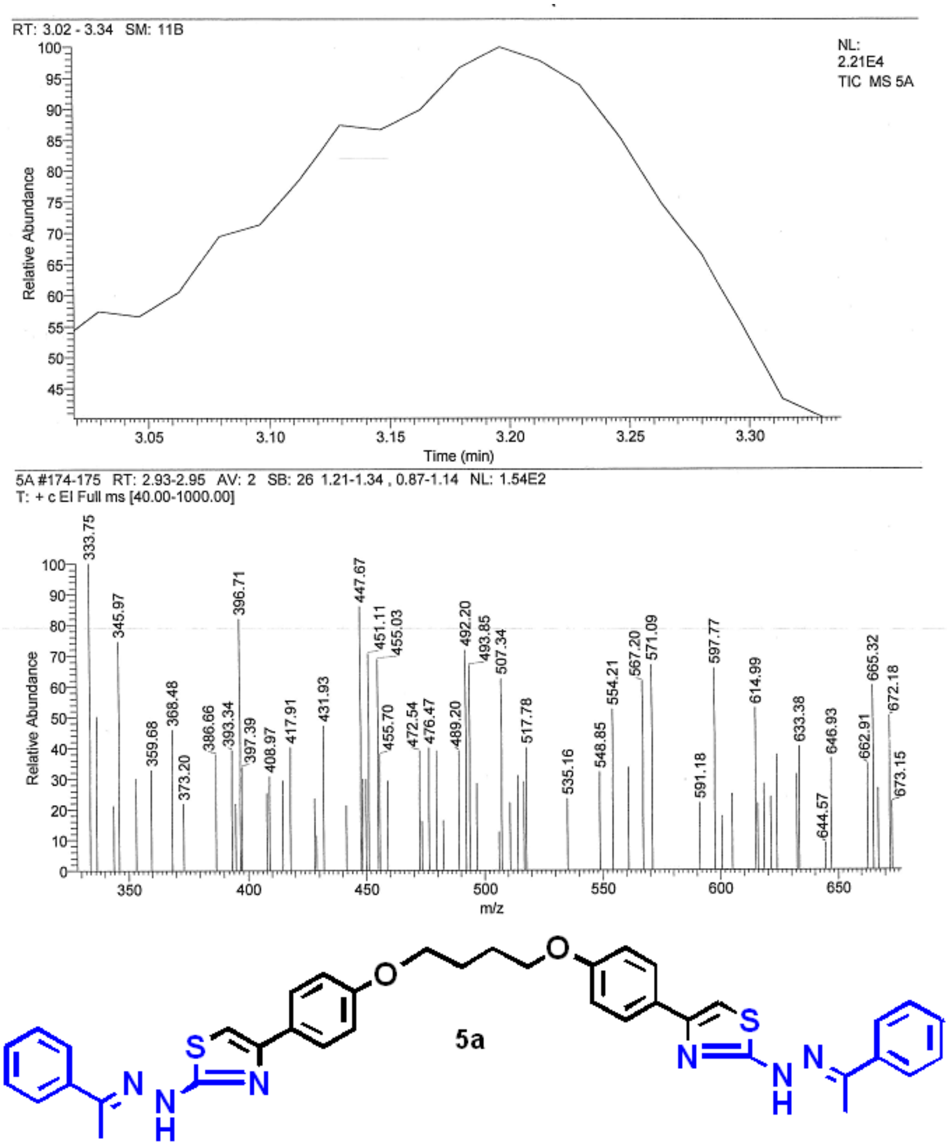

Mass spectrum compound 5a

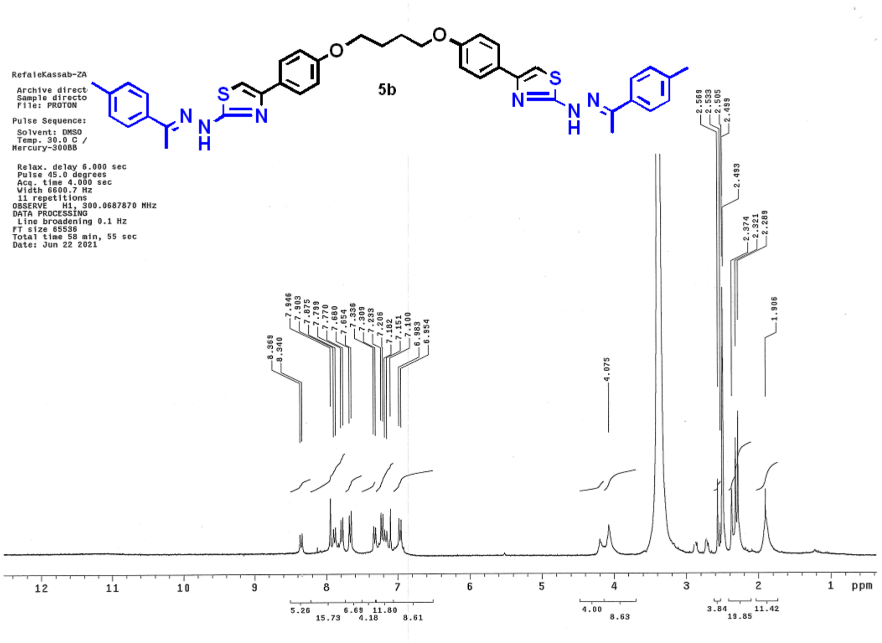

^{1}H NMR spectrum compound 5b

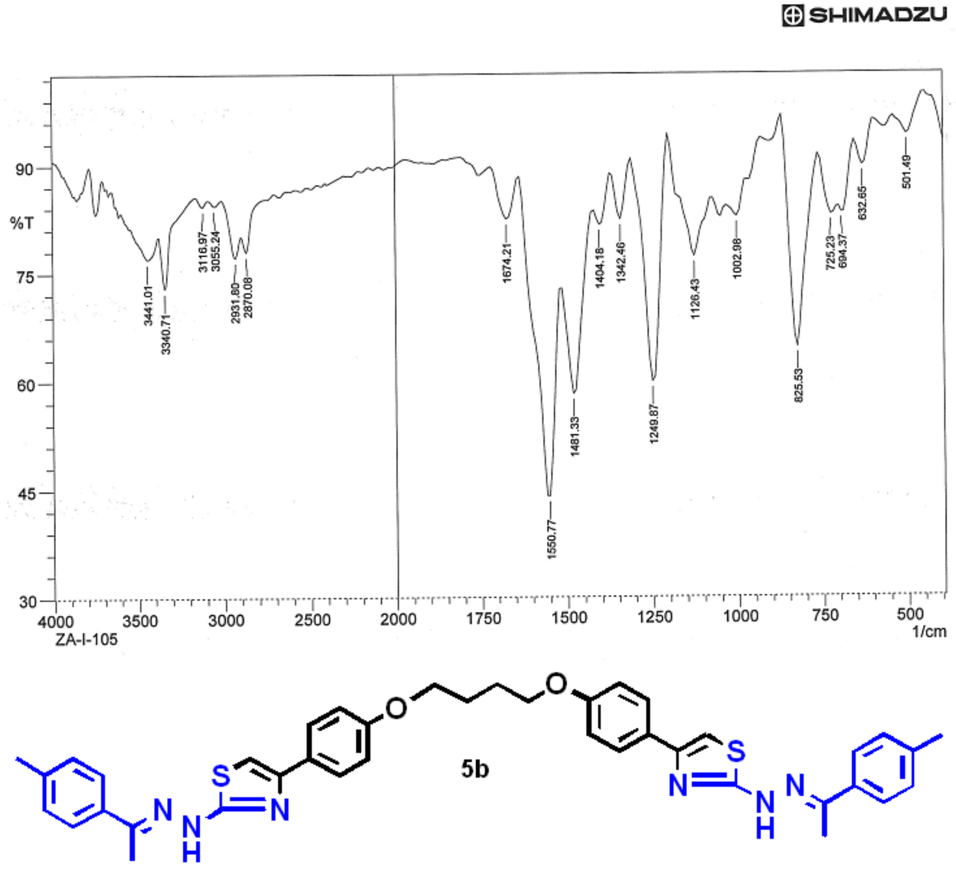

IR spectrum compound 5b

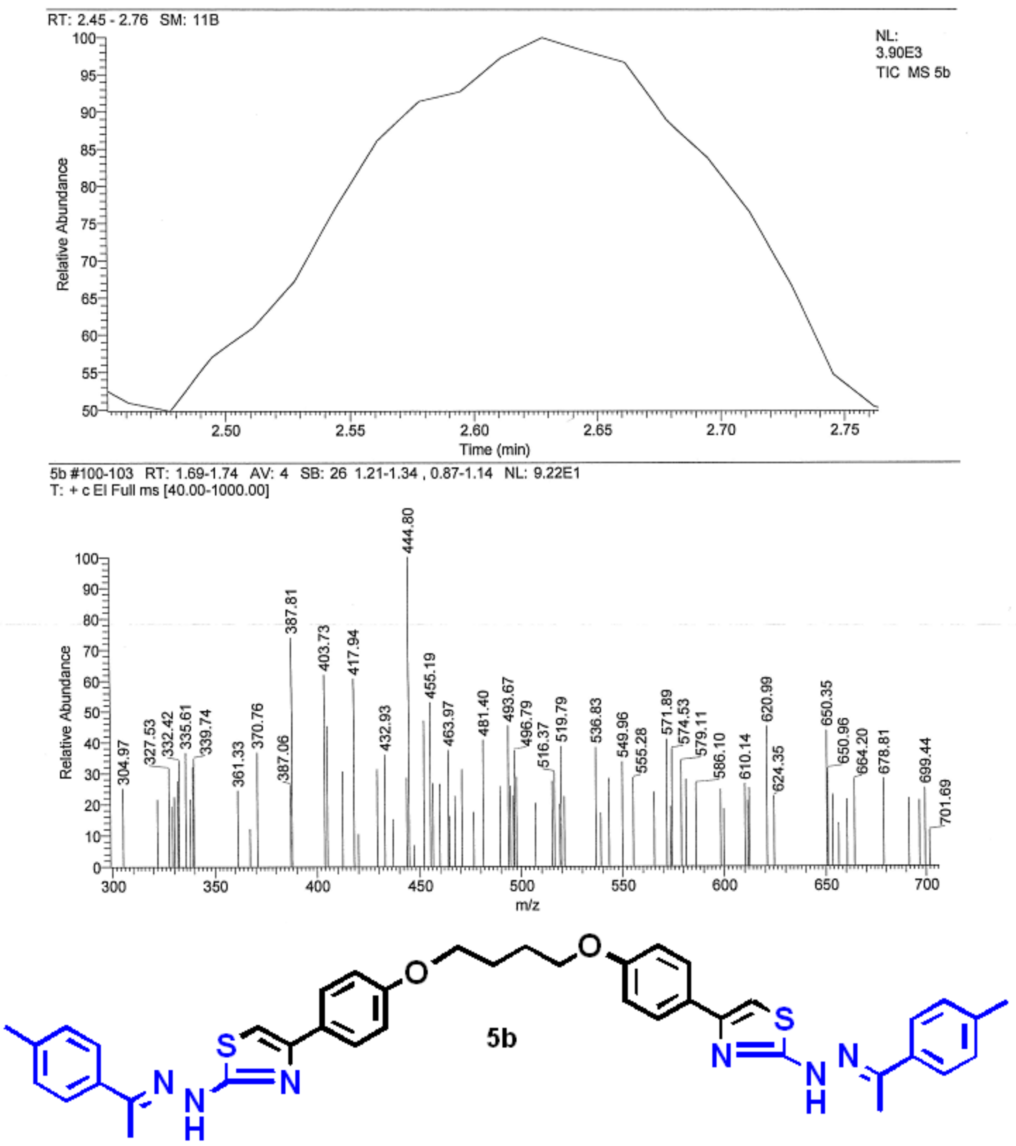

Mass spectrum compound 5a

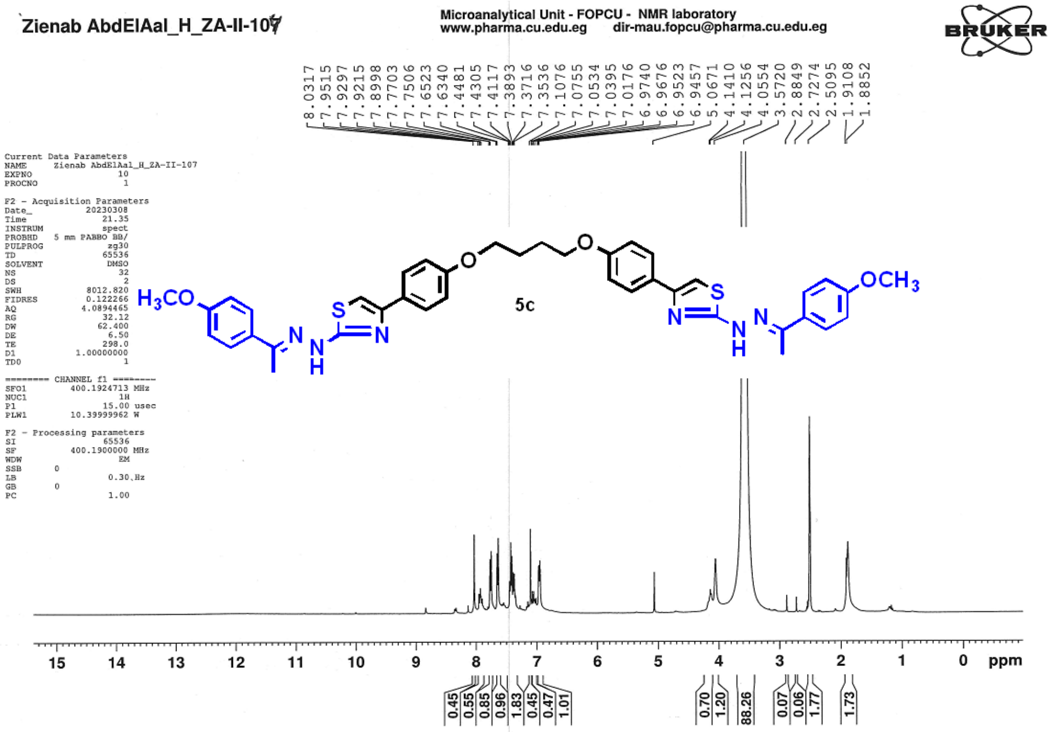

^{1}H NMR spectrum compound 5c

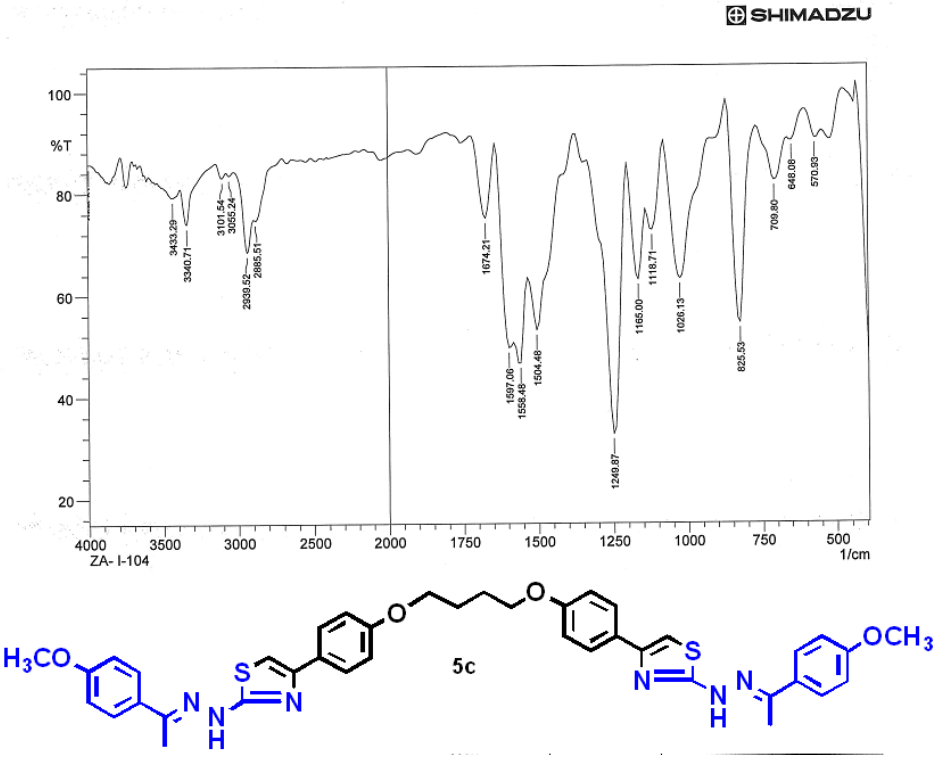

IR spectrum compound 5c

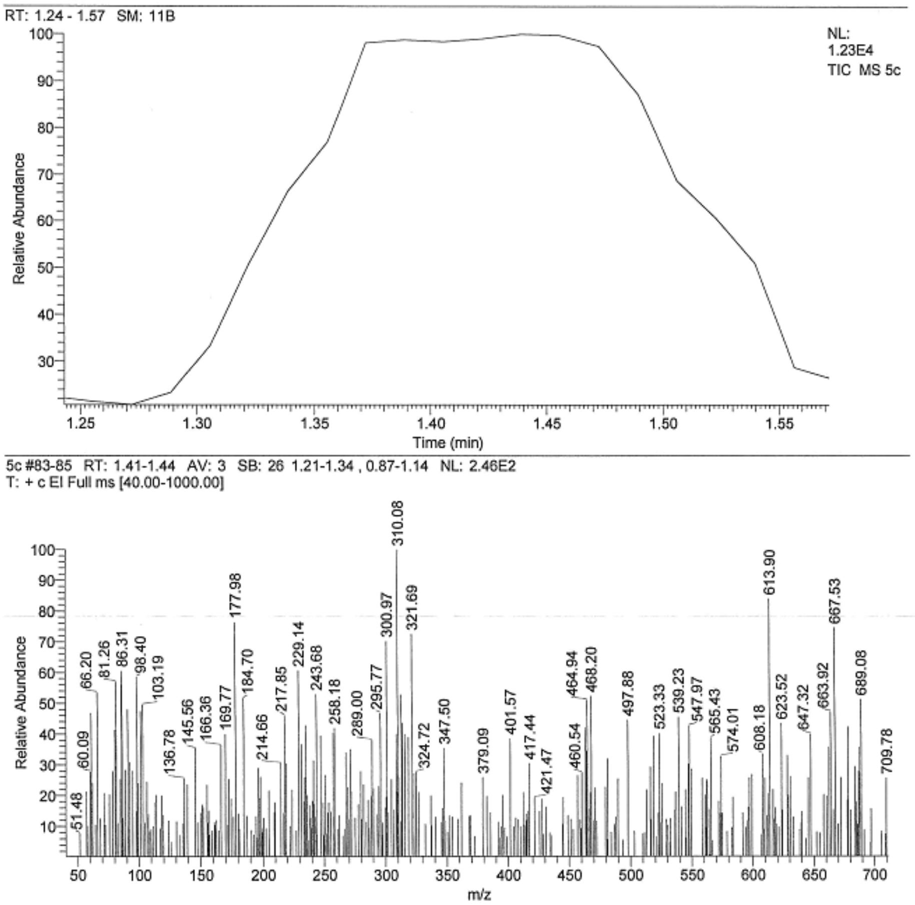

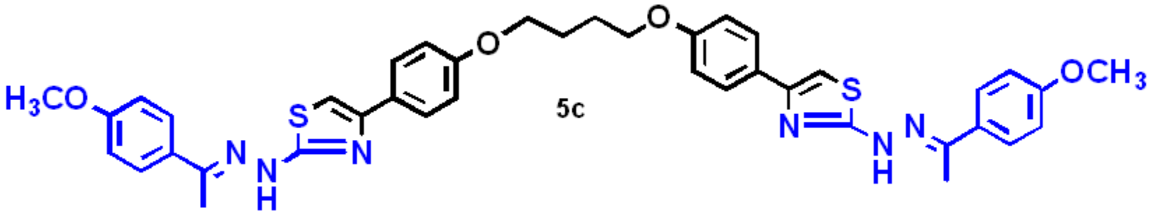

Mass spectrum compound 5c

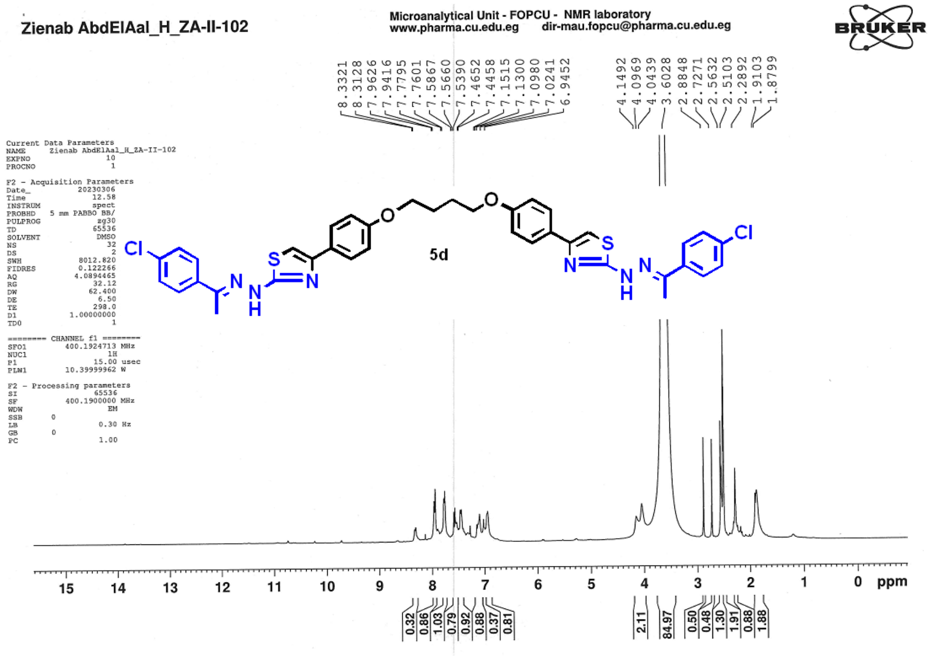

^{1}H NMR spectrum compound 5d

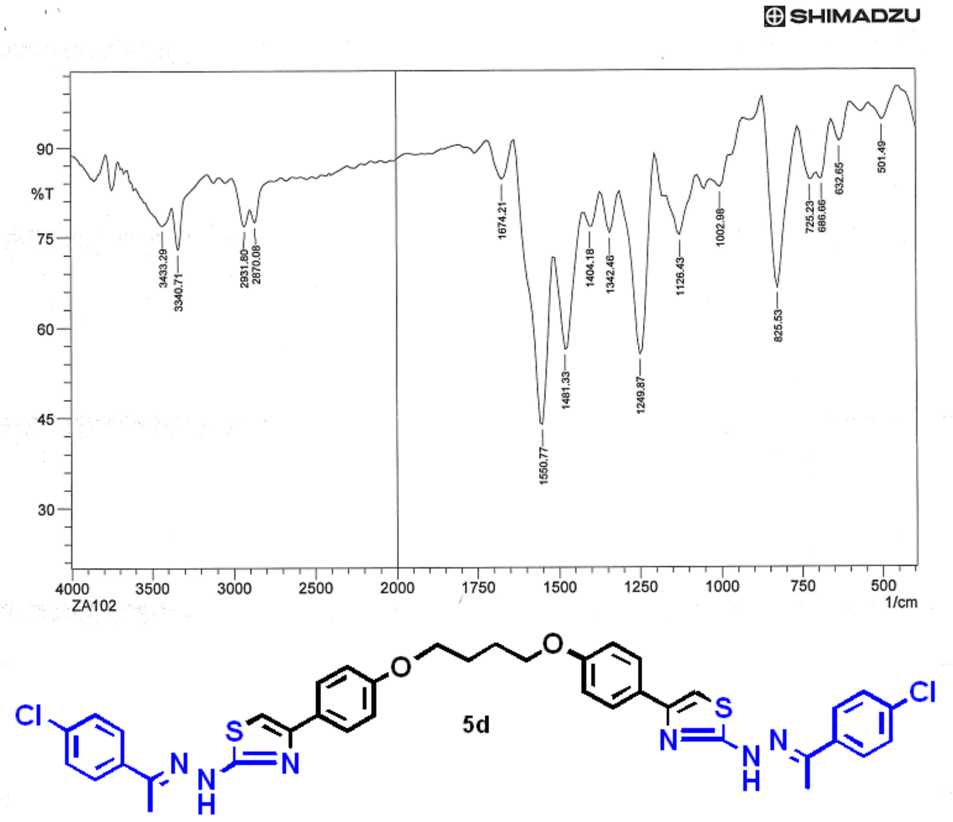

IR spectrum compound 5d

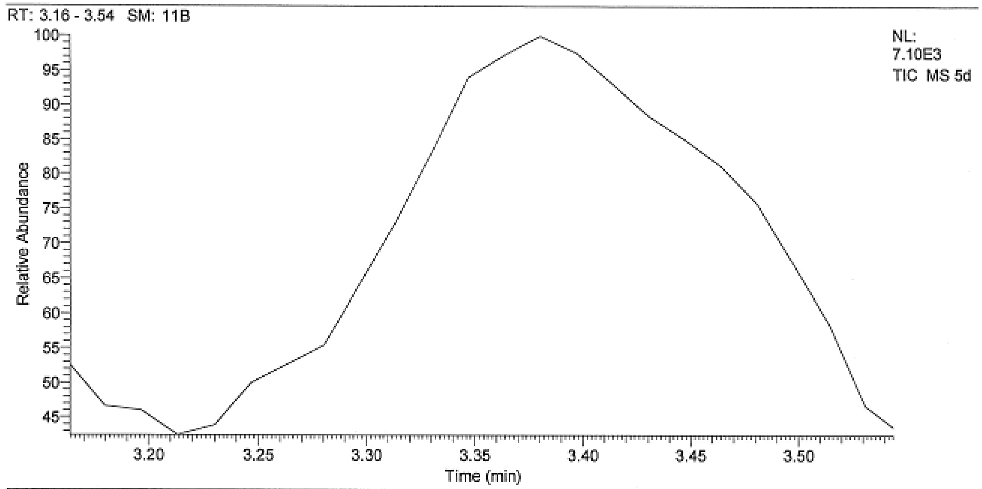

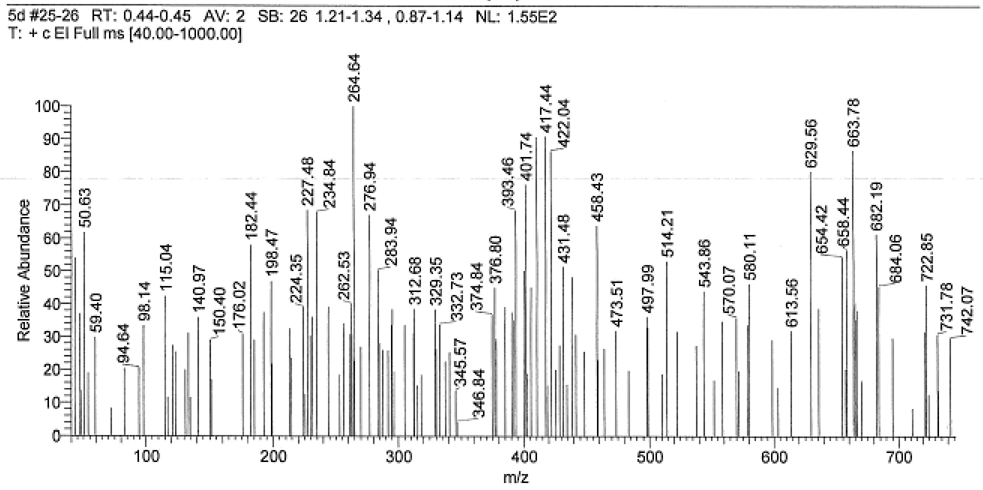

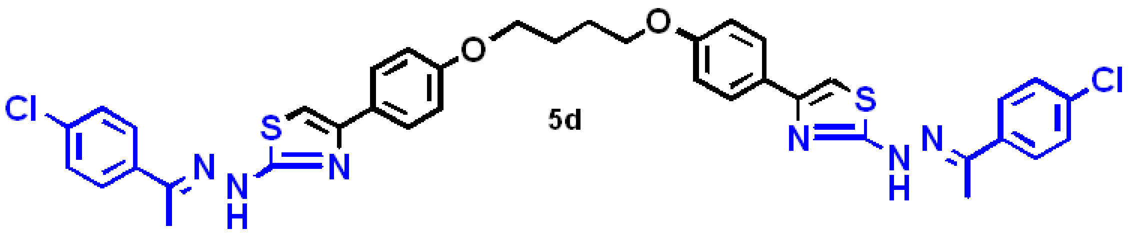

Mass spectrum compound 5d

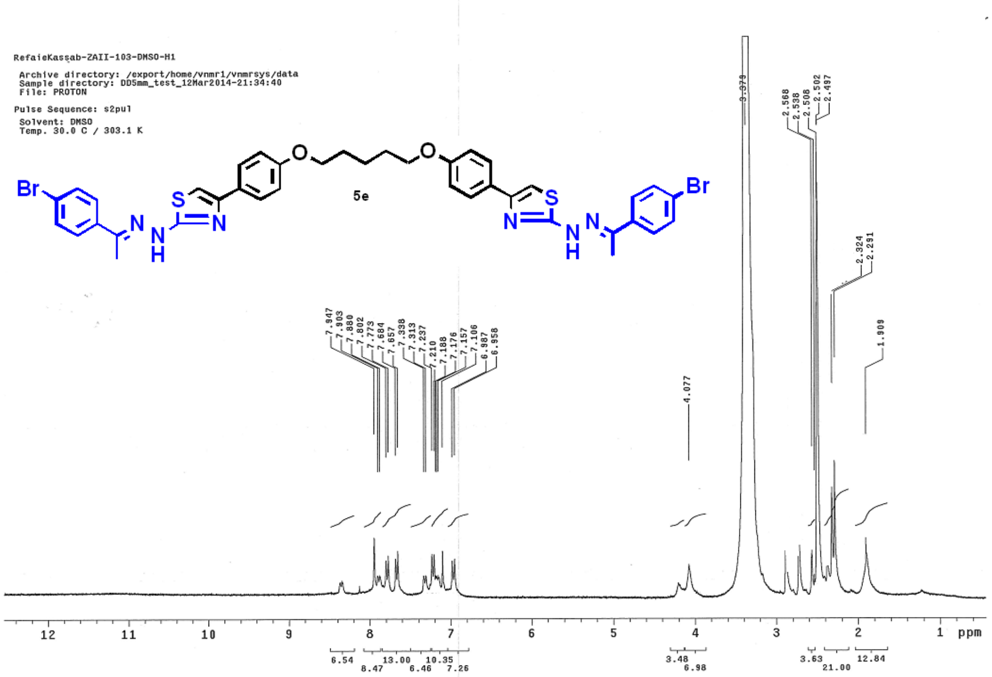

^{1}H NMR spectrum compound 5e

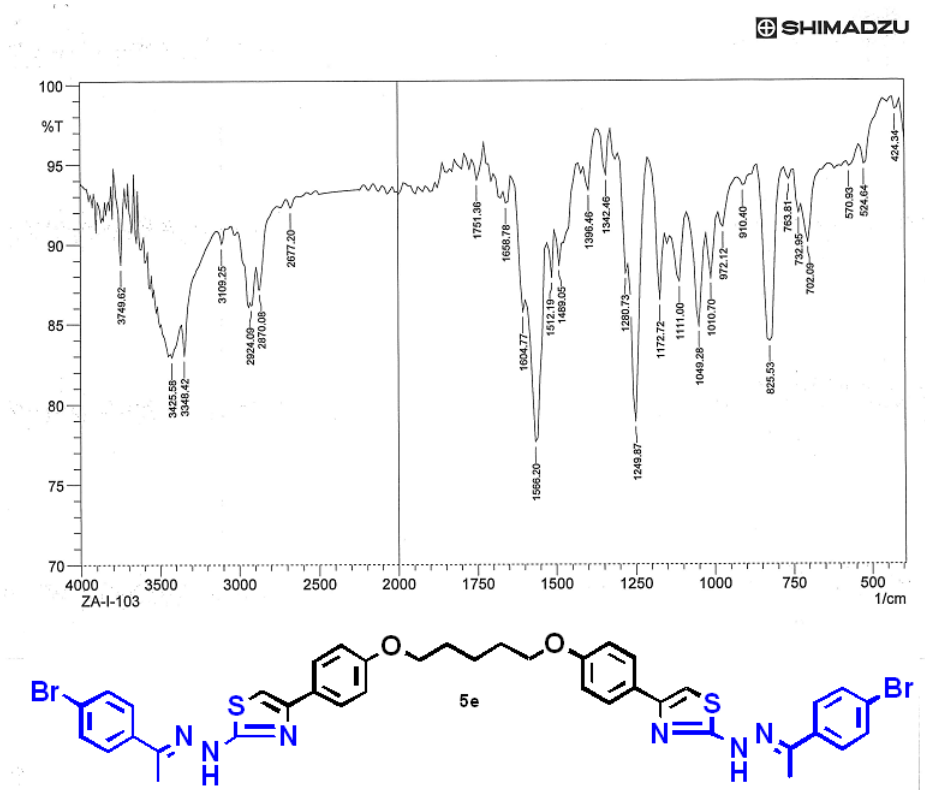

IR spectrum compound 5e

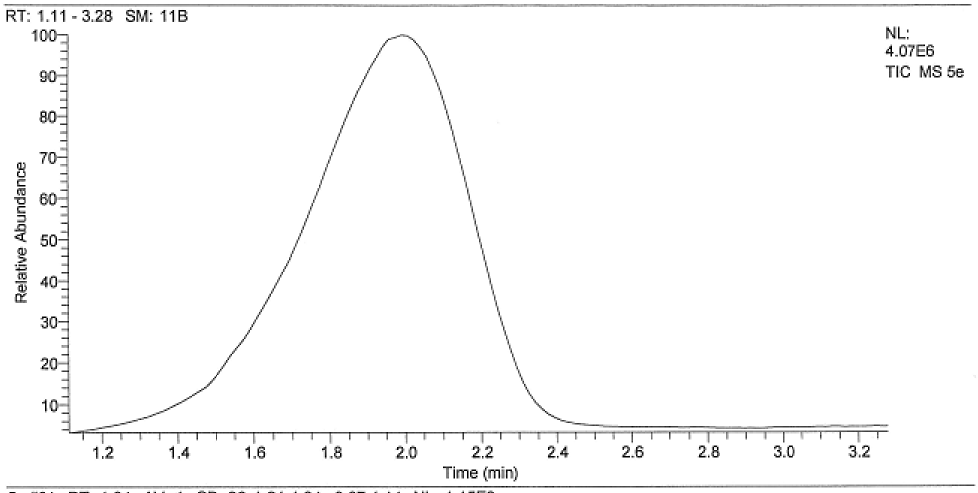

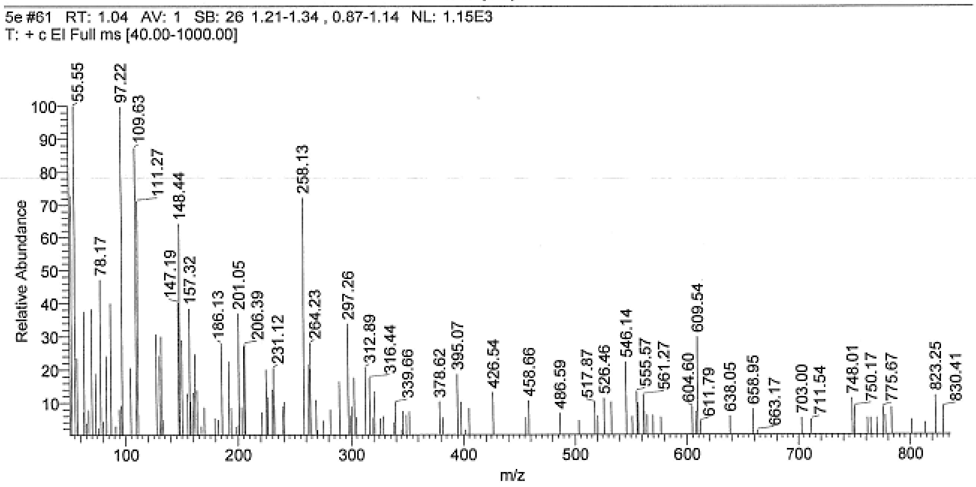

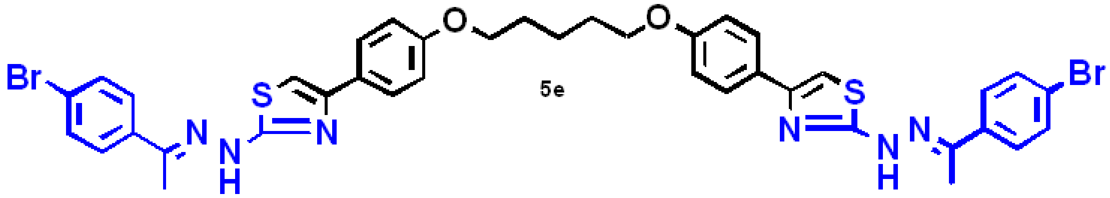

Mass spectrum compound 5e

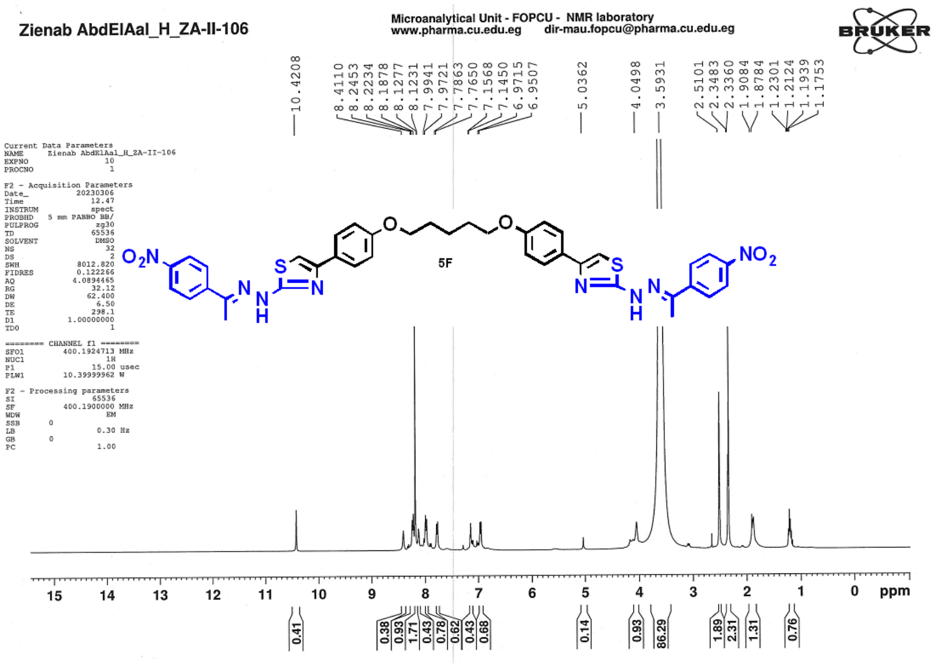

^{1}H NMR spectrum compound 5f

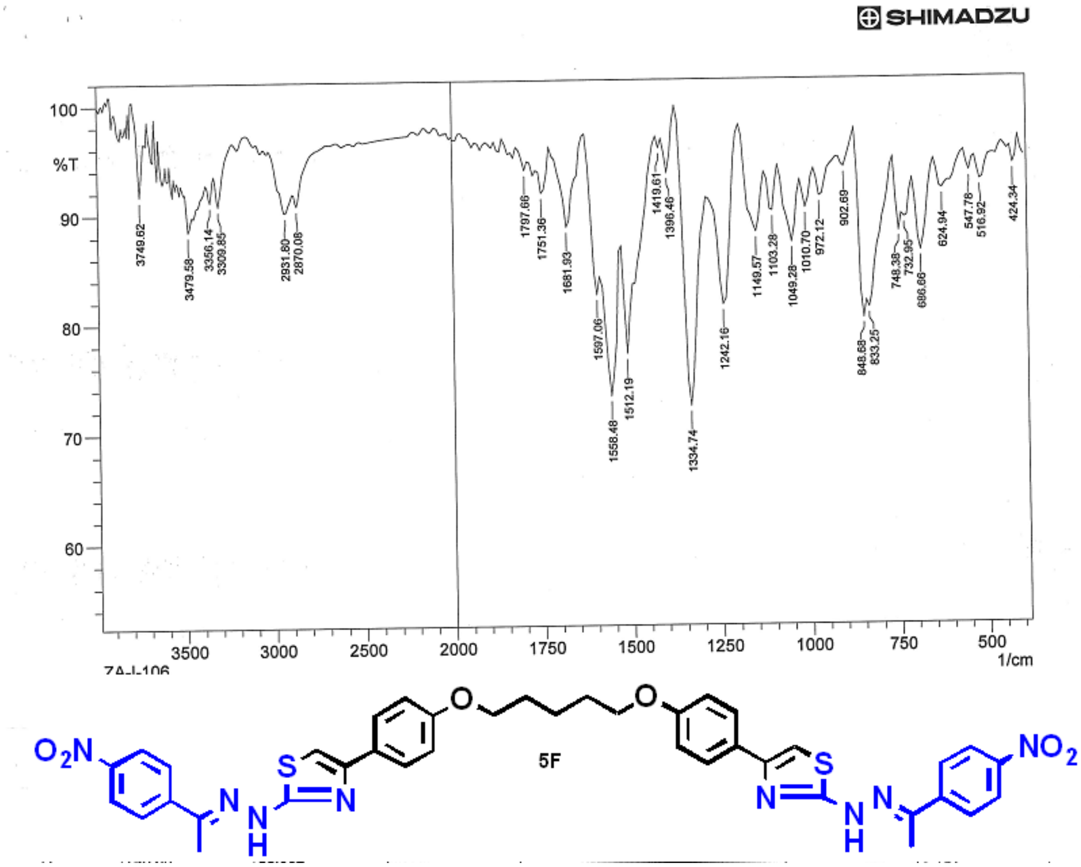

IR spectrum compound 5f

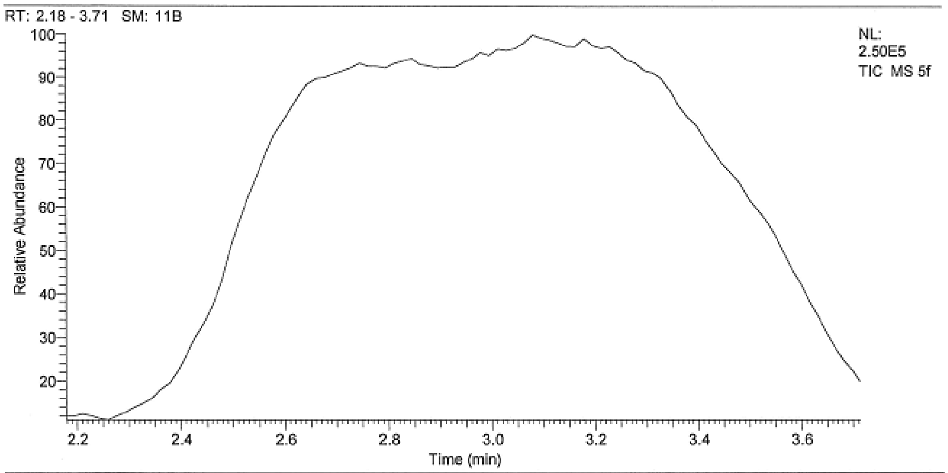

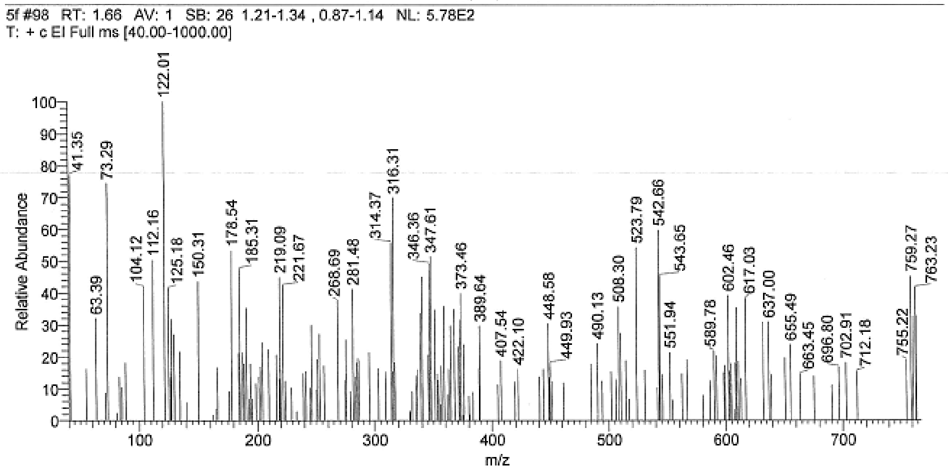

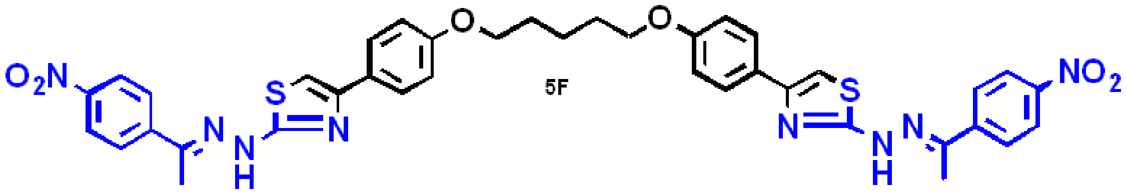

Mass spectrum compound 5f

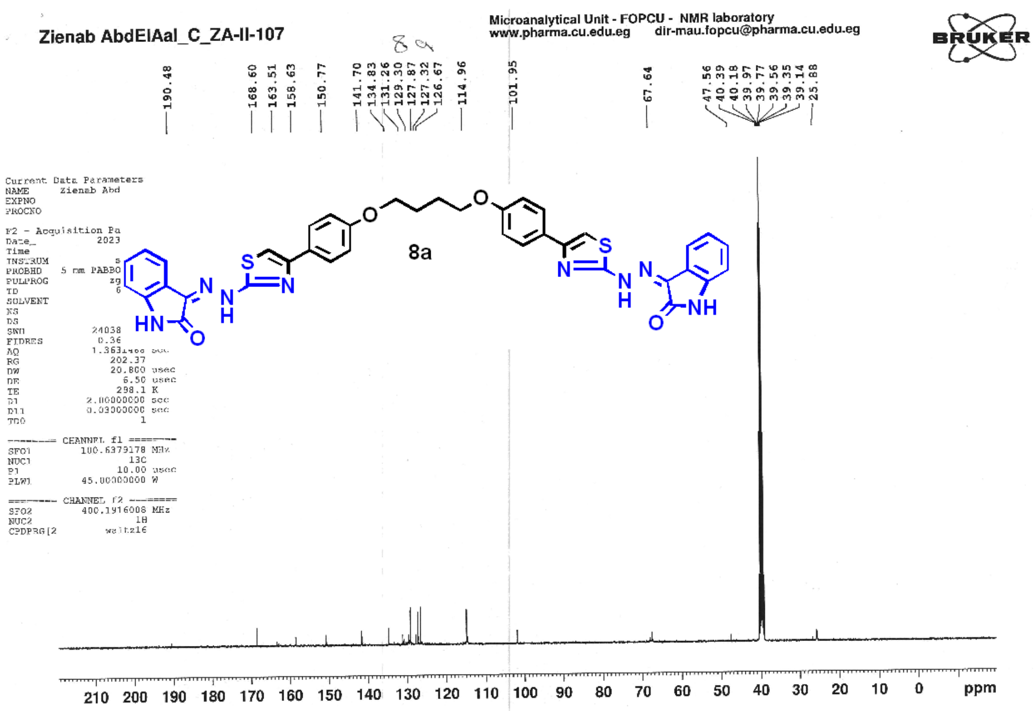

^{13}C NMR spectrum compound 8a

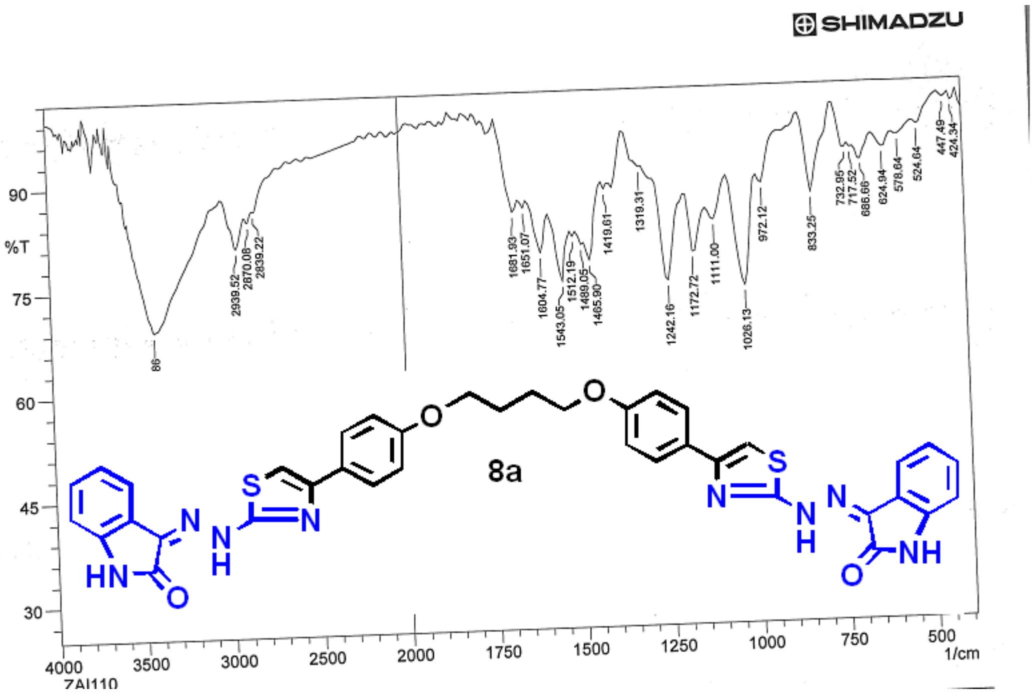

IR spectrum compound 8a

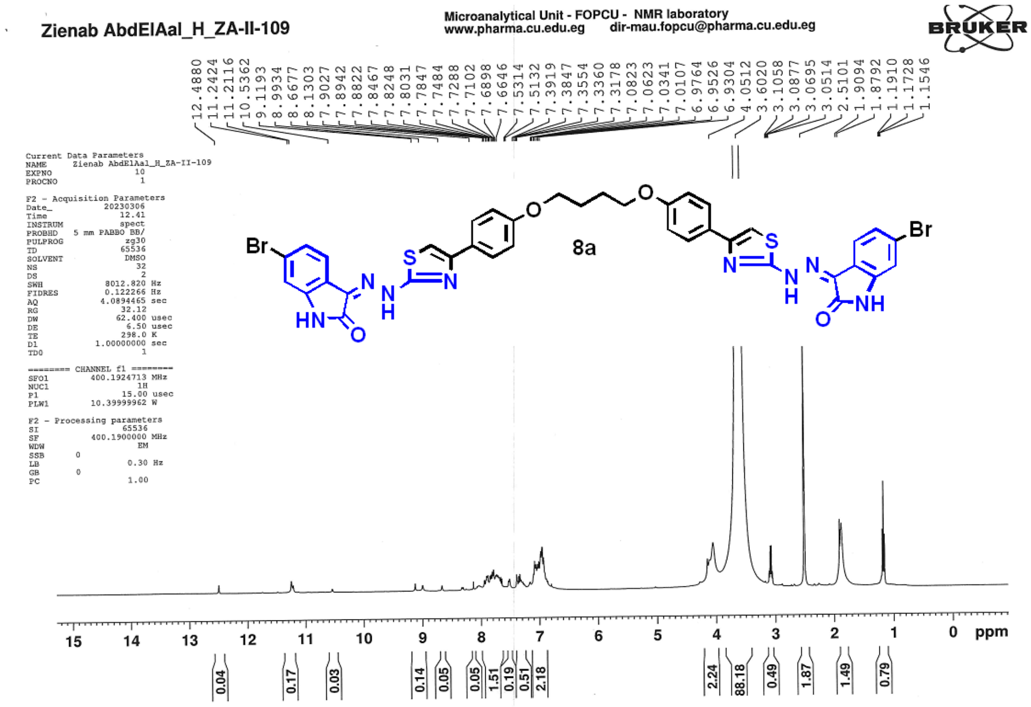

^{1}H NMR spectrum compound 8b

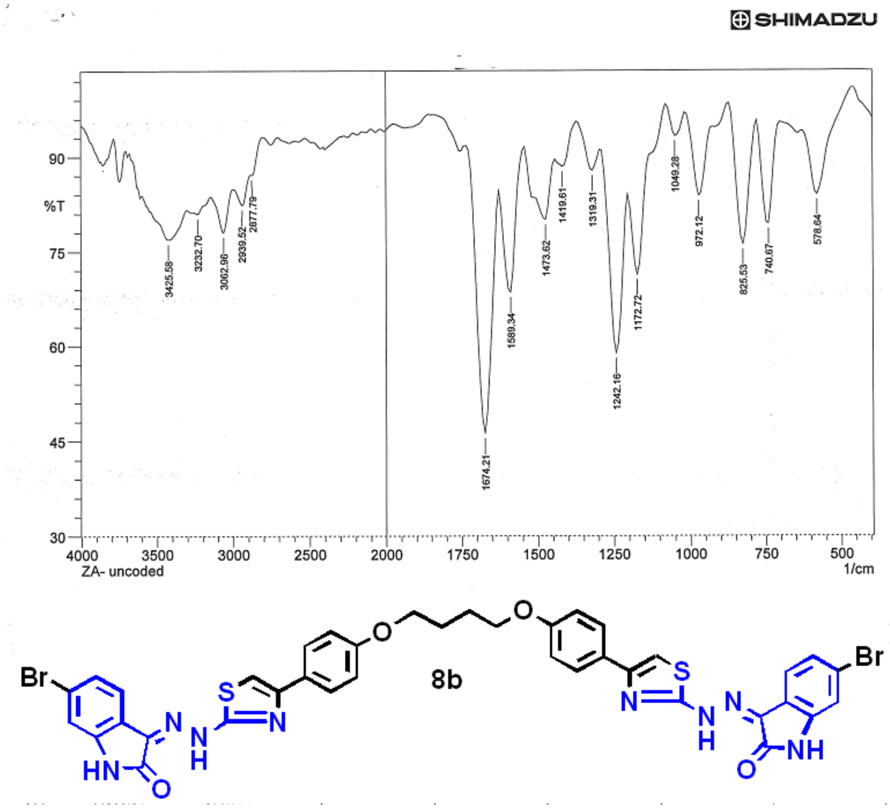

IR spectrum compound 8b

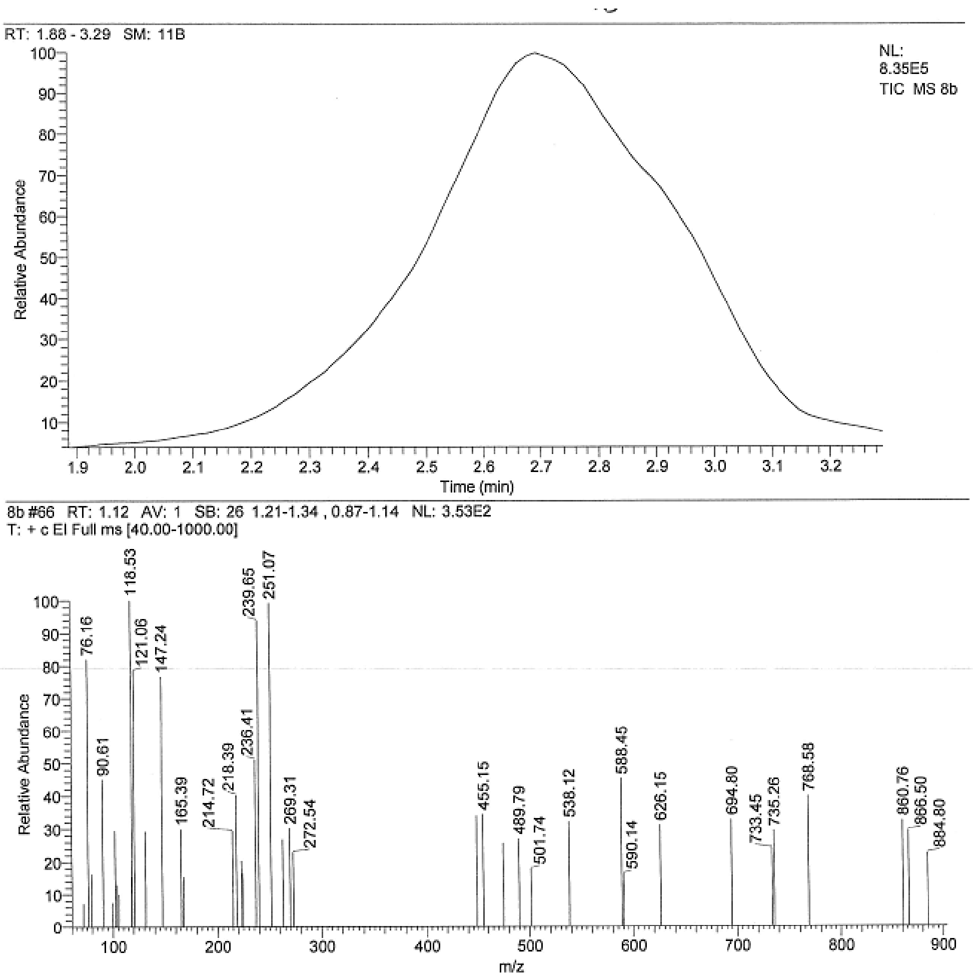

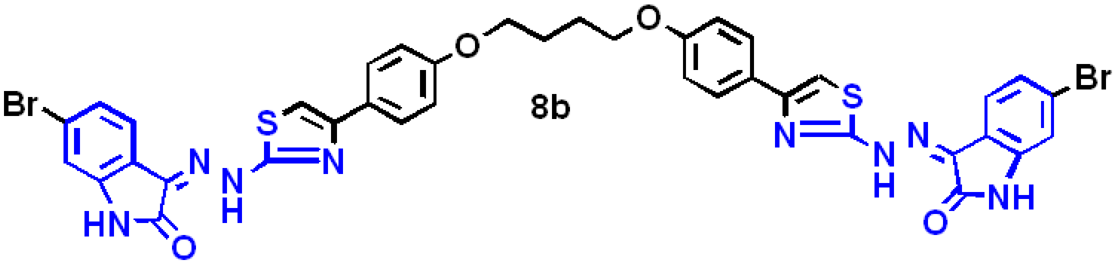

8b

Mass spectrum compound 8b

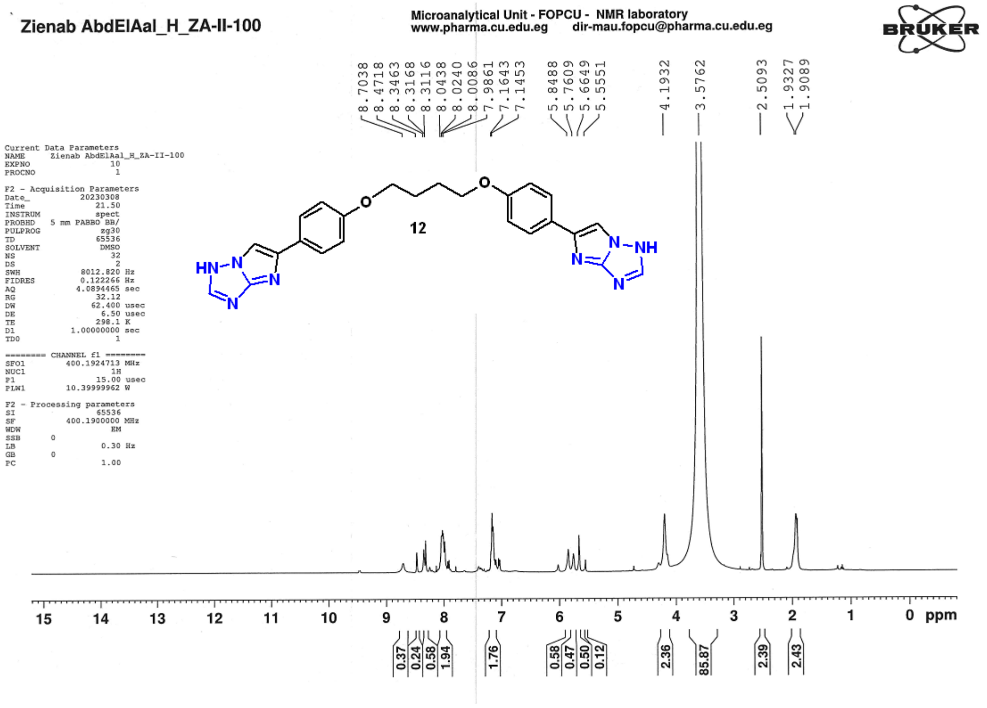

^{1}H NMR spectrum compound 12

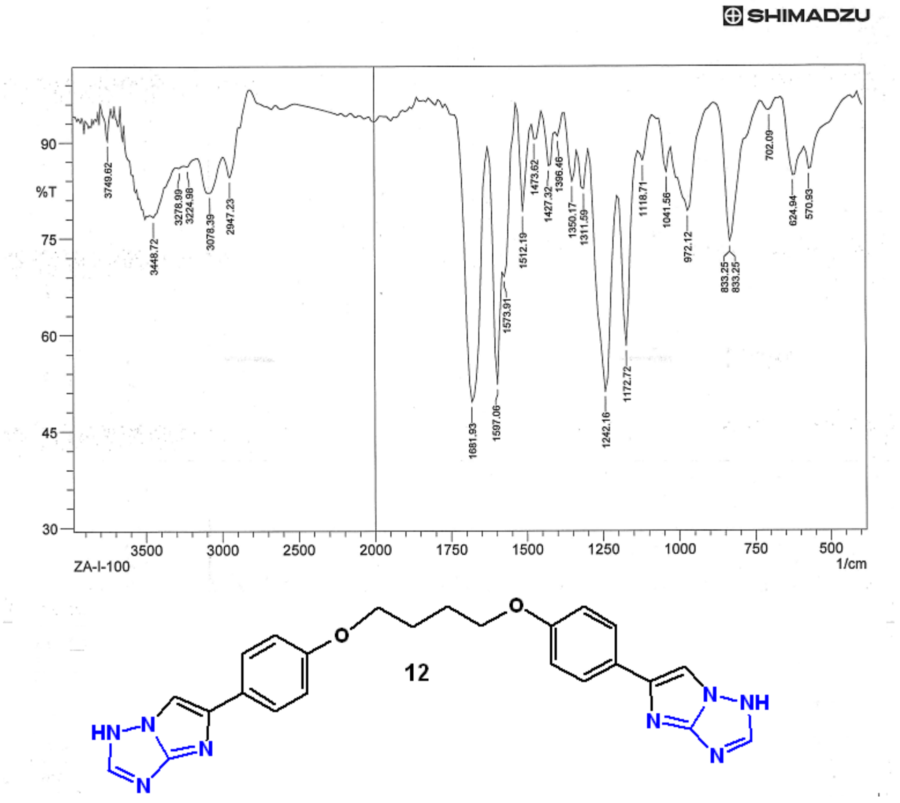

IR spectrum compound 12

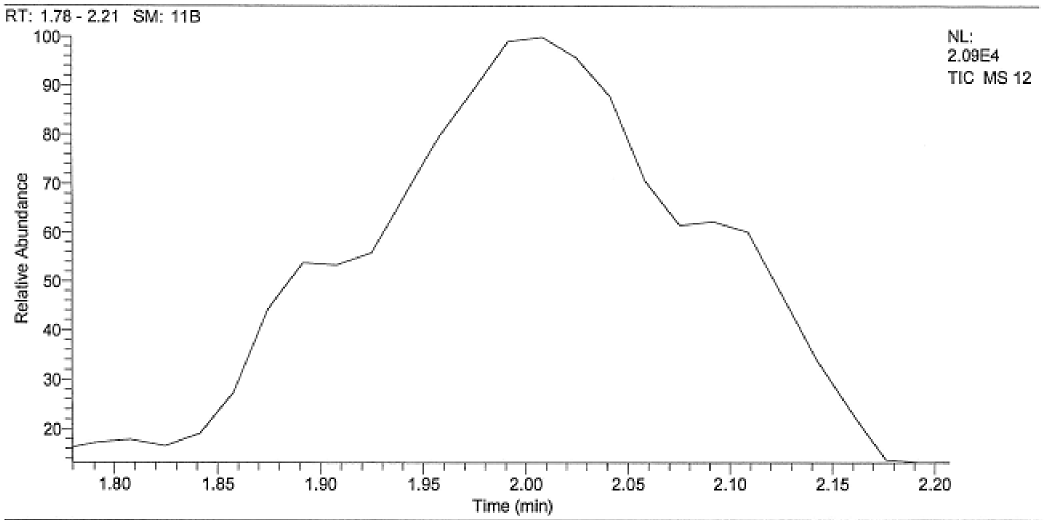

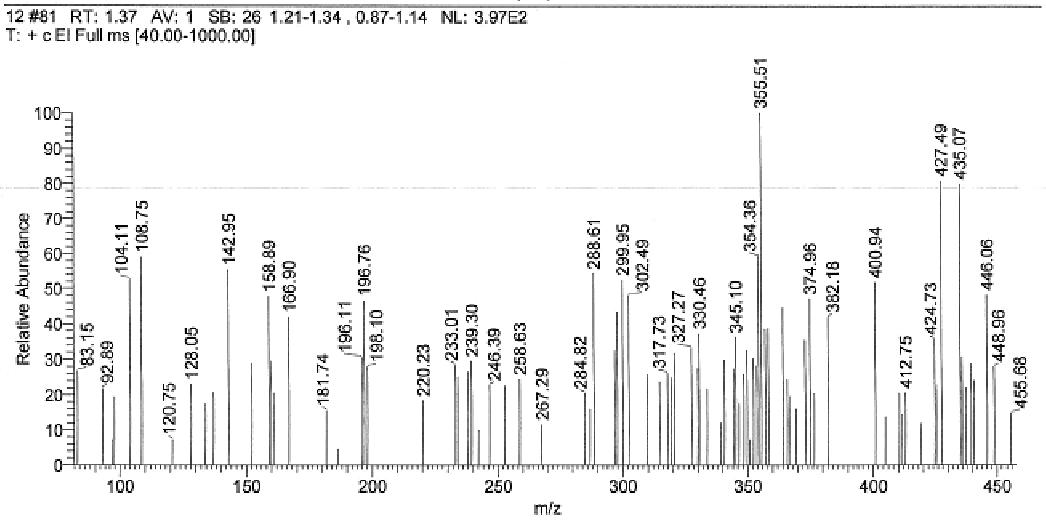

m/z

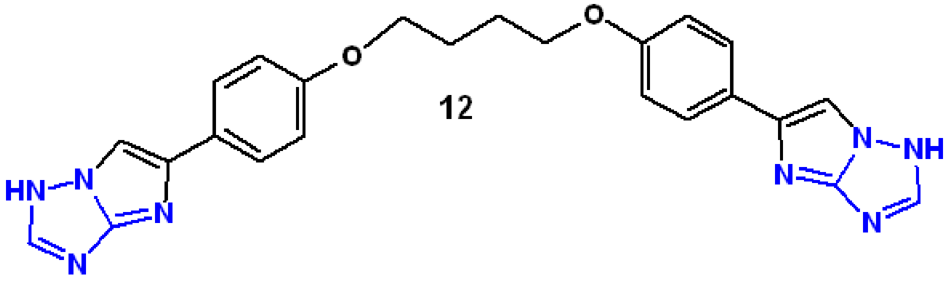

Mass spectrum compound 12

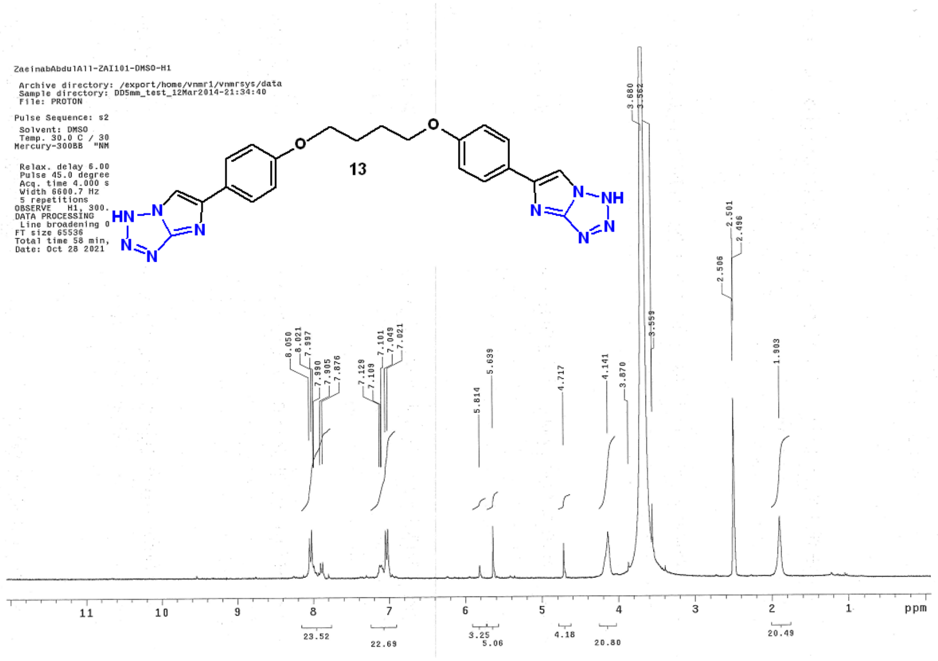

^{1}H NMR spectrum compound 13

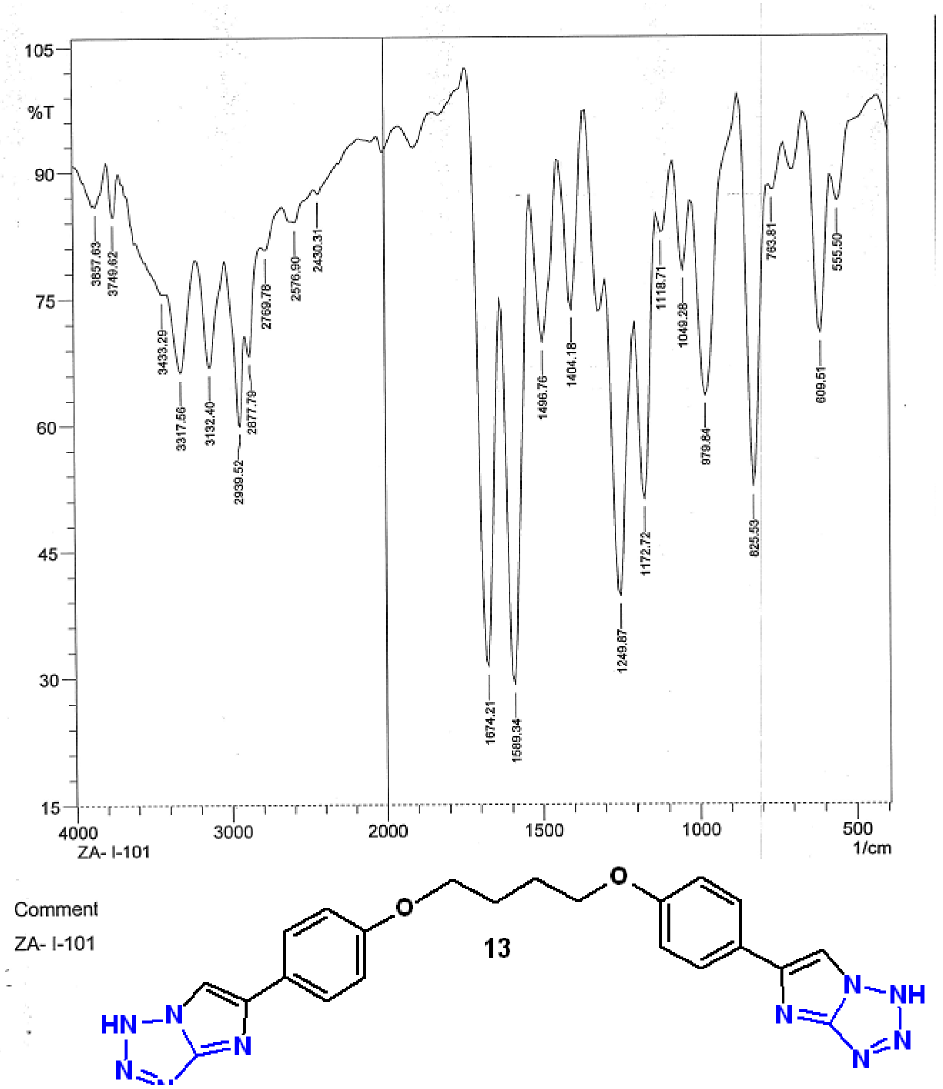

IR spectrum compound 13

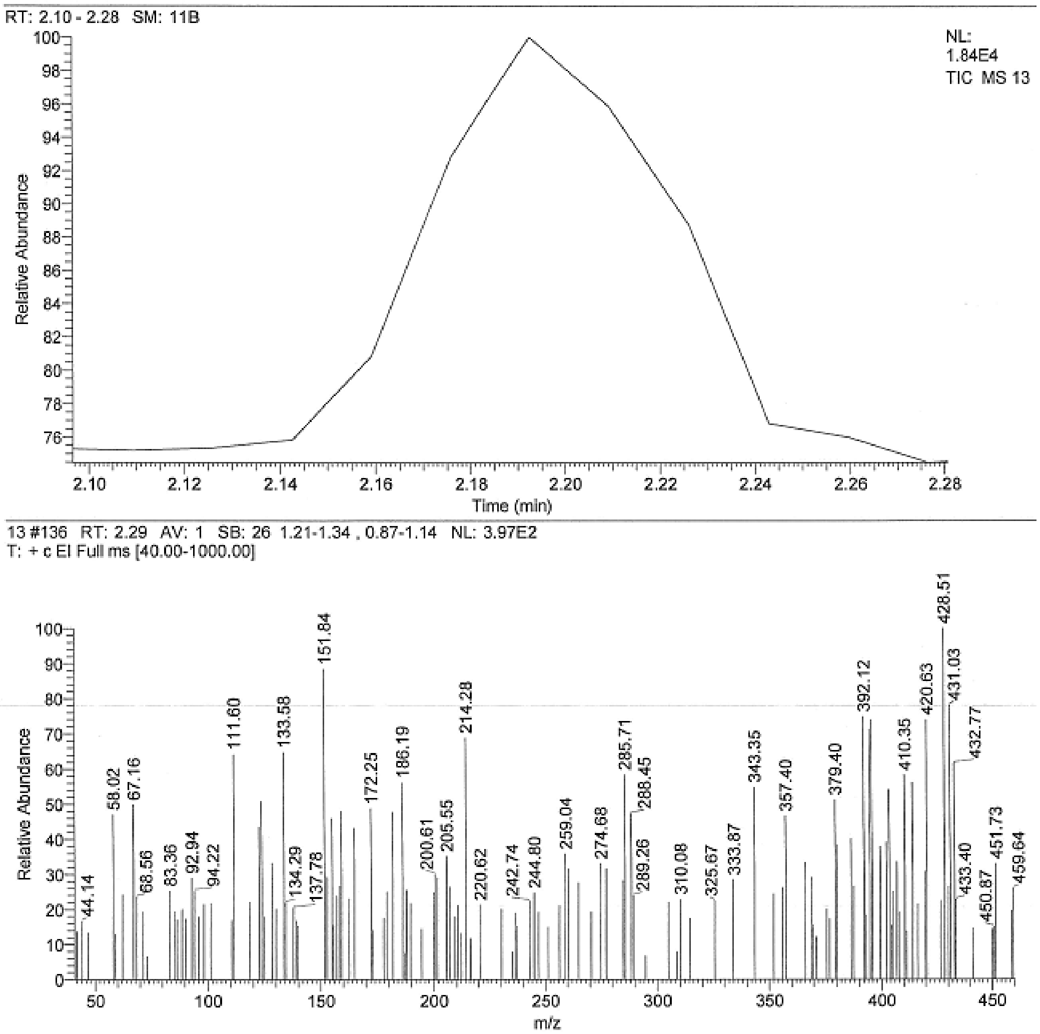

m/z Ir

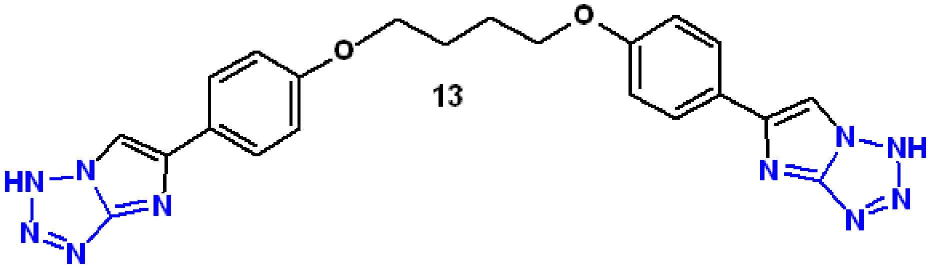

Mass spectrum compound 13

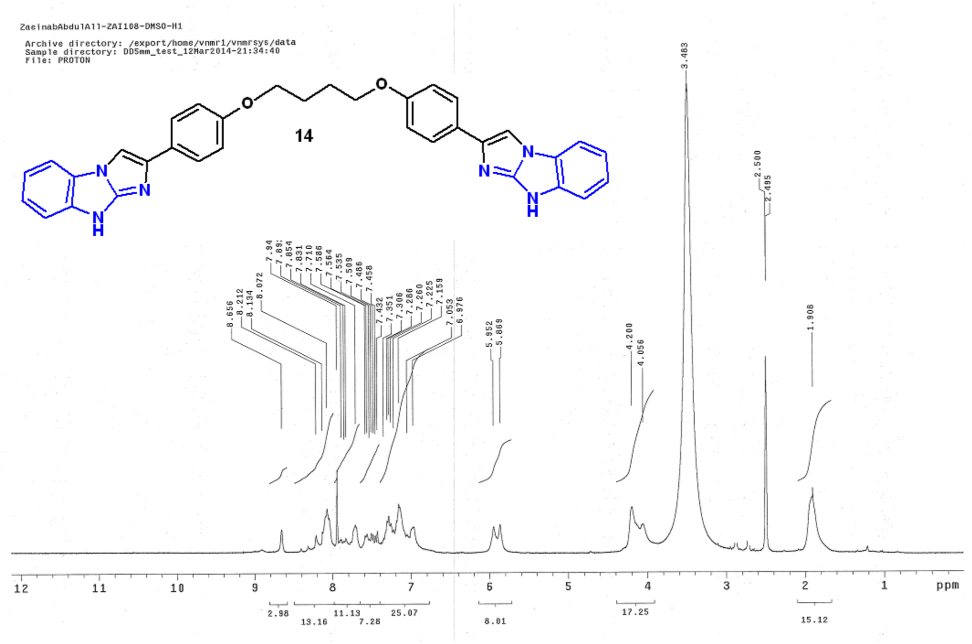

^{1}H NMR spectrum compound 14

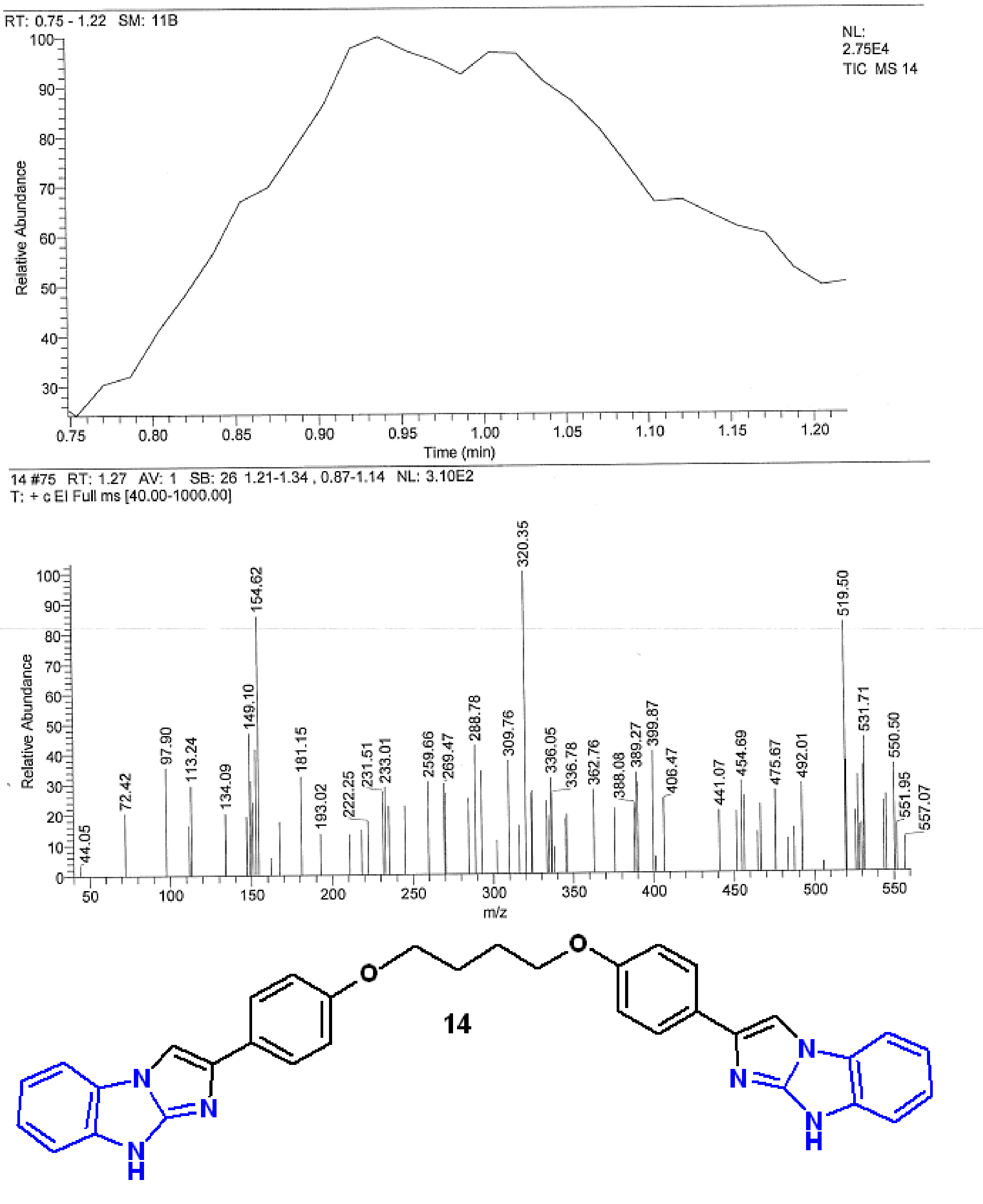

Mass spectrum compound 14

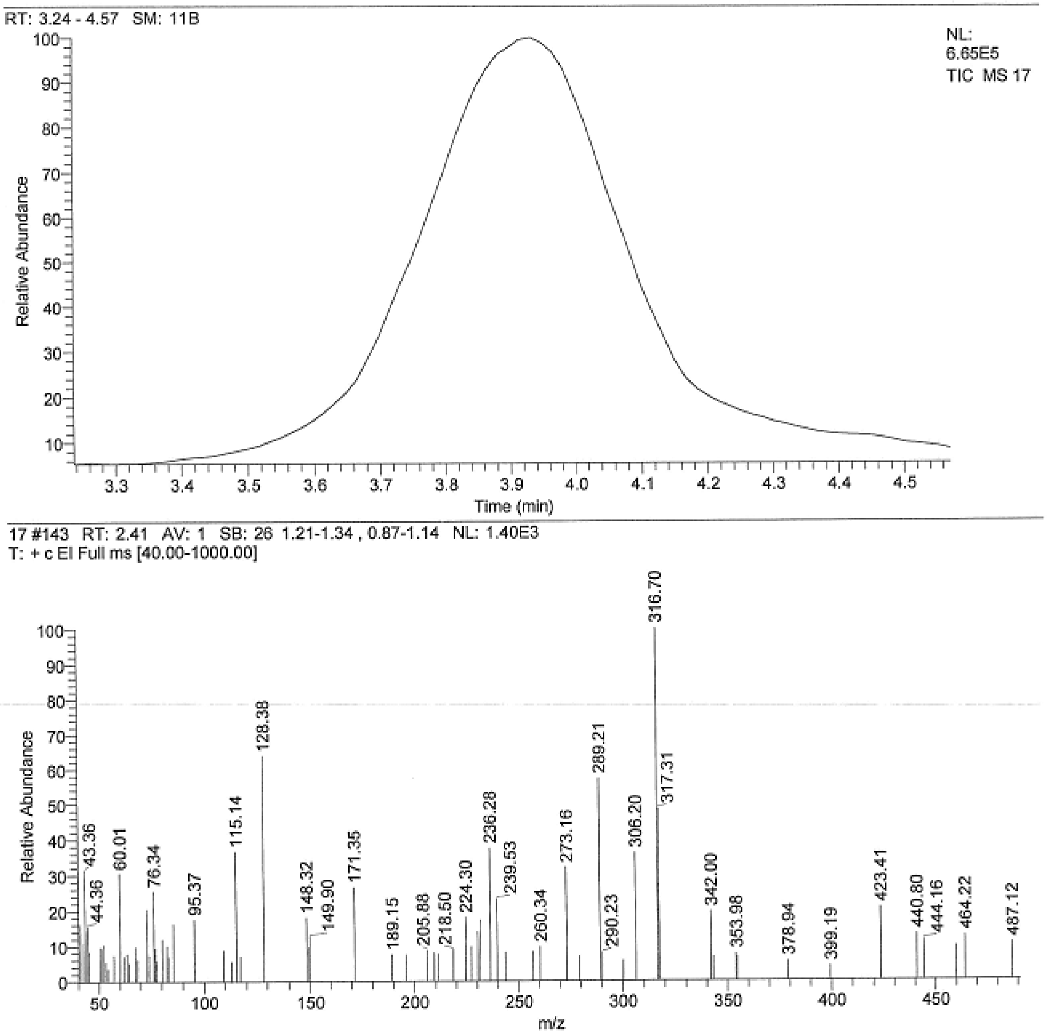

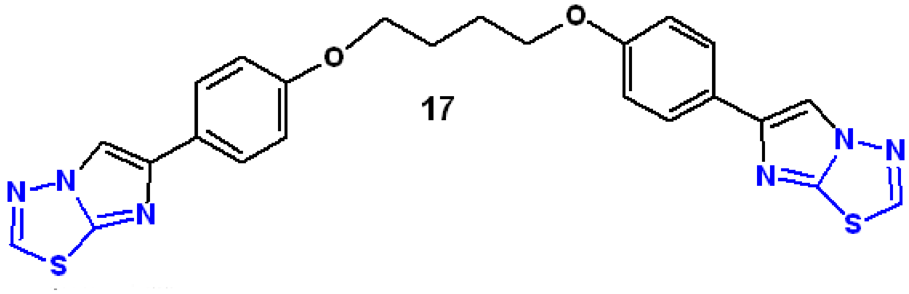

Mass spectrum compound 17

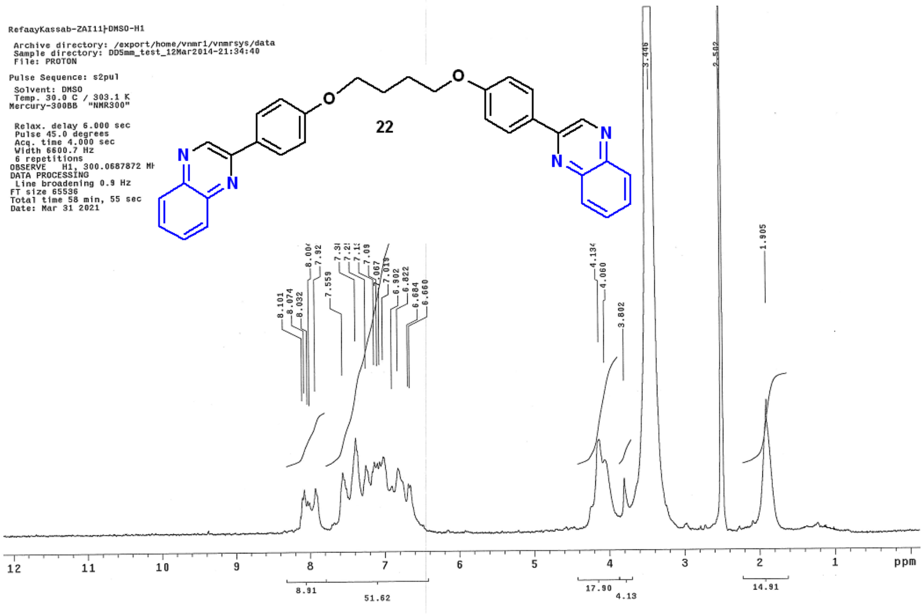

^{1}H NMR spectrum compound 22

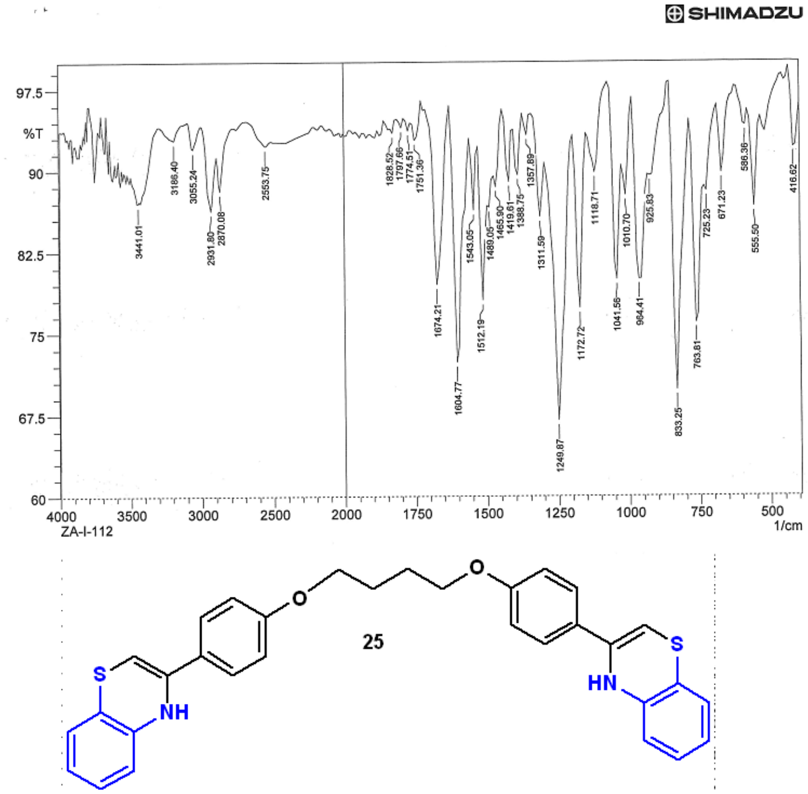

IR spectrum compound 25

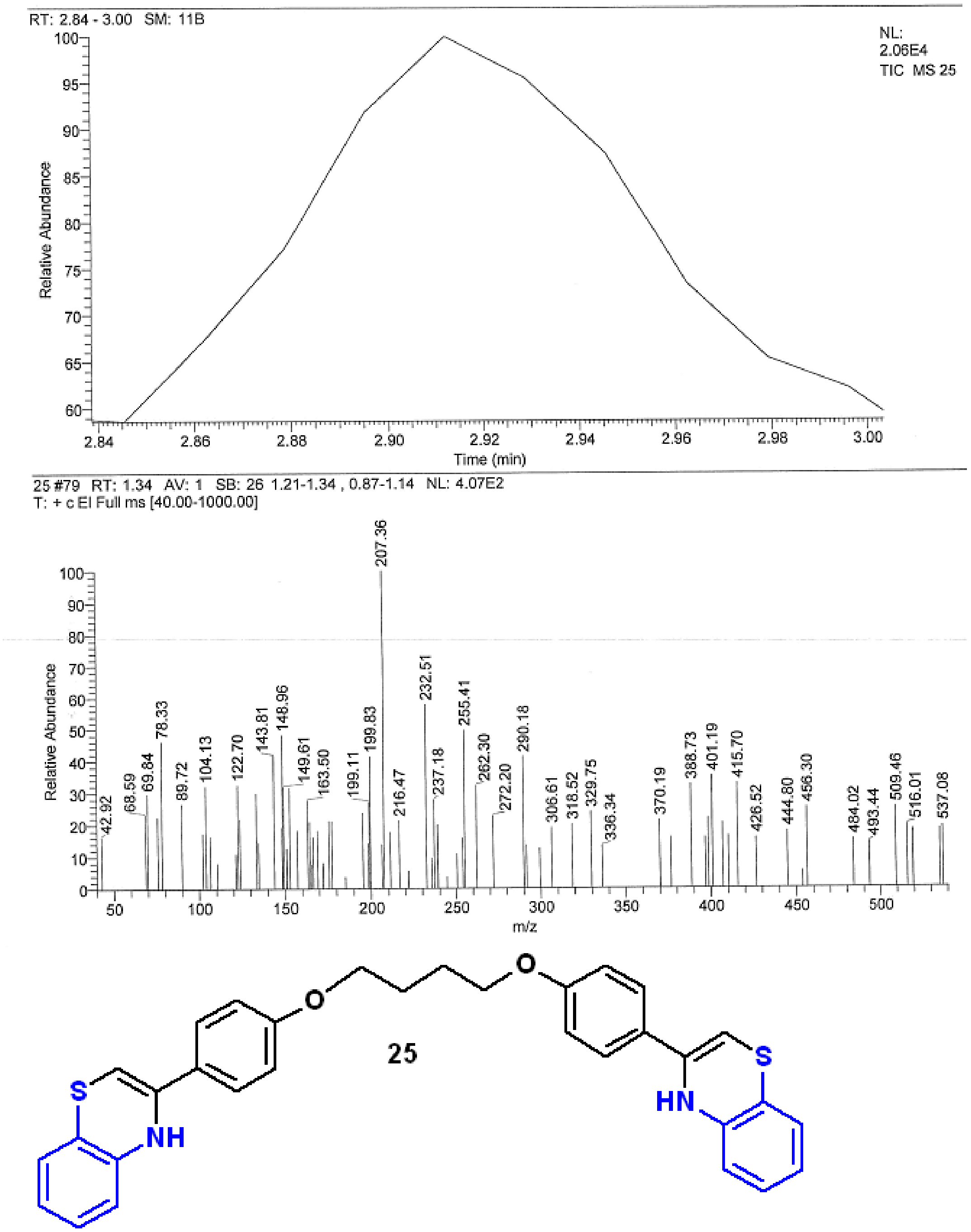

Mass spectrum compound 25

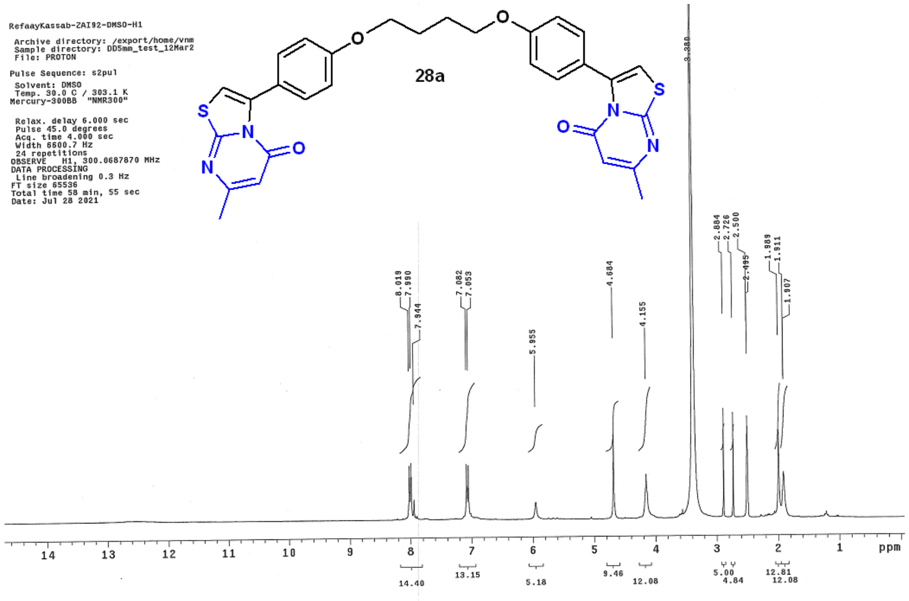

^{1}H NMR spectrum compound 28a

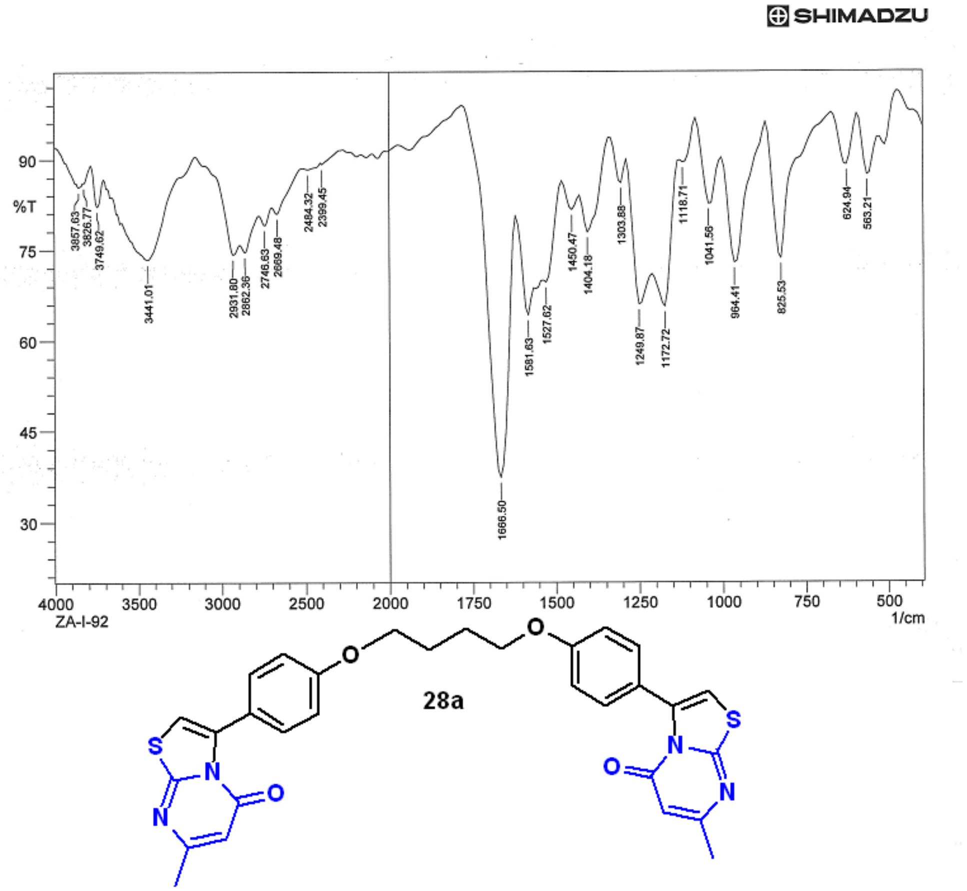

IR spectrum compound 28a

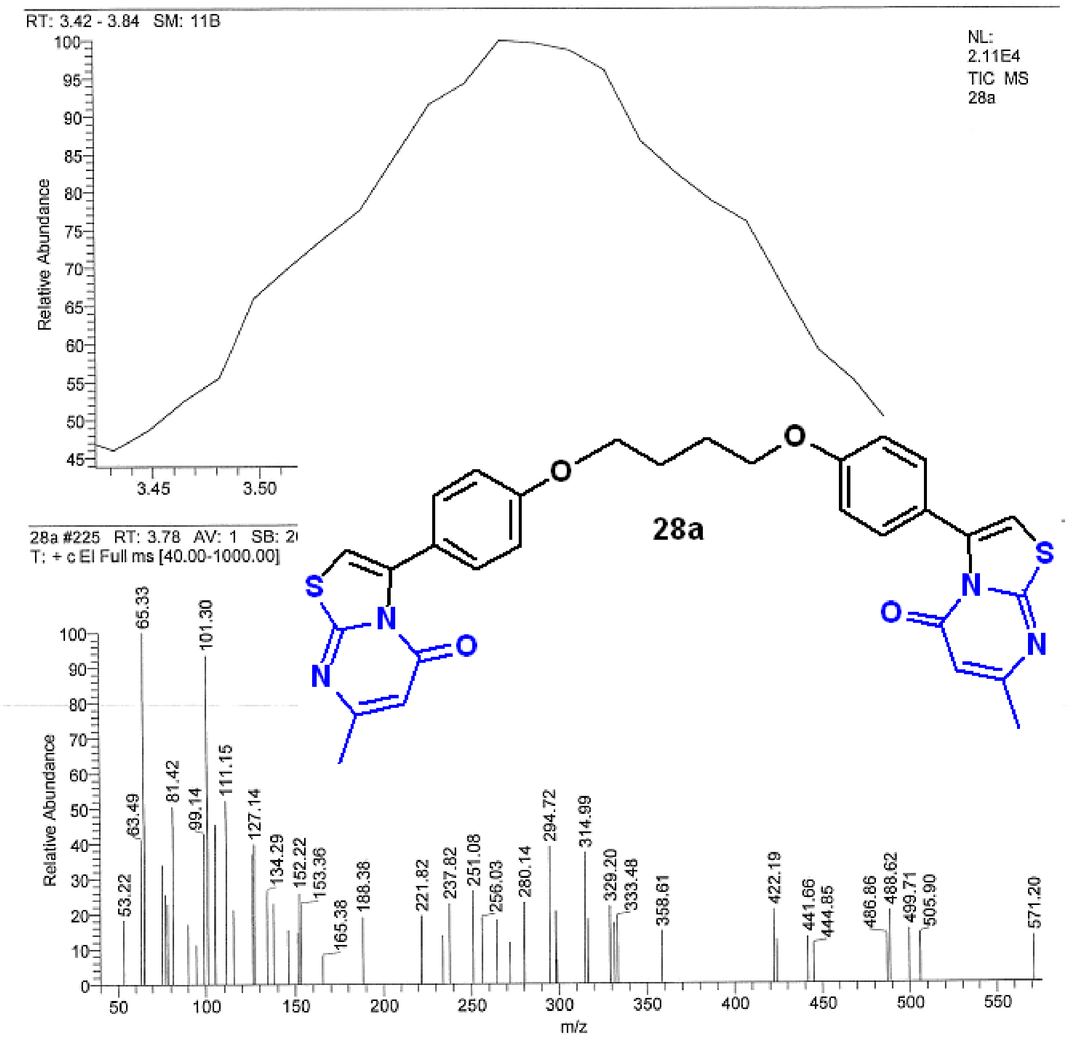

Mass spectrum compound 28a

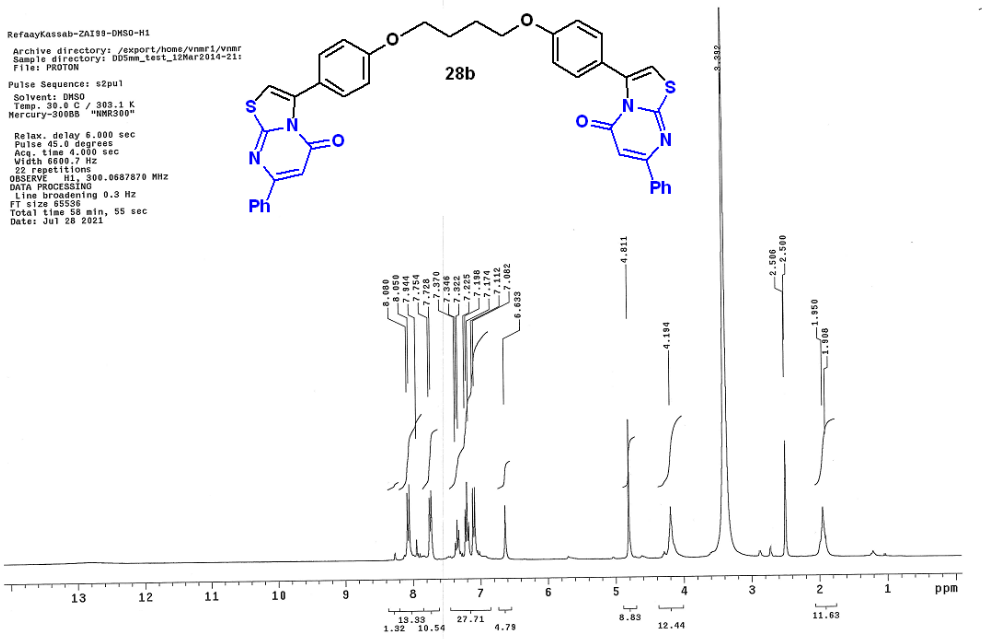

^{1}H NMR spectrum compound 28b

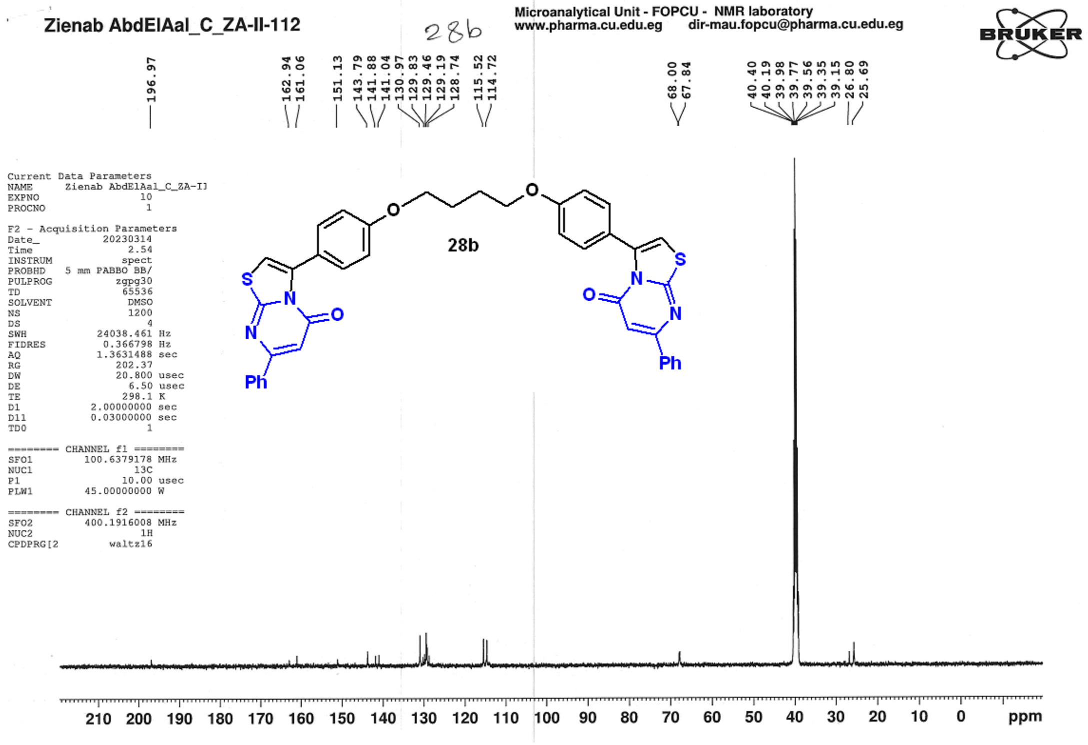

^{13}C NMR spectrum compound 28b

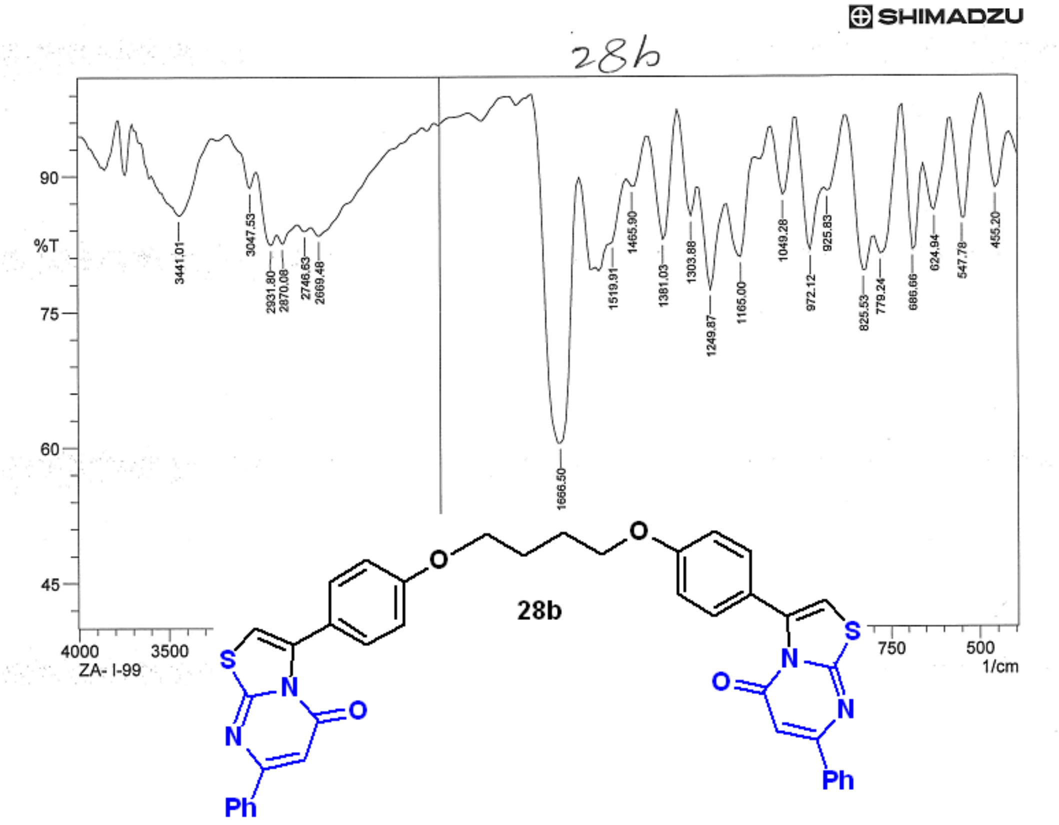

IR spectrum compound 28b

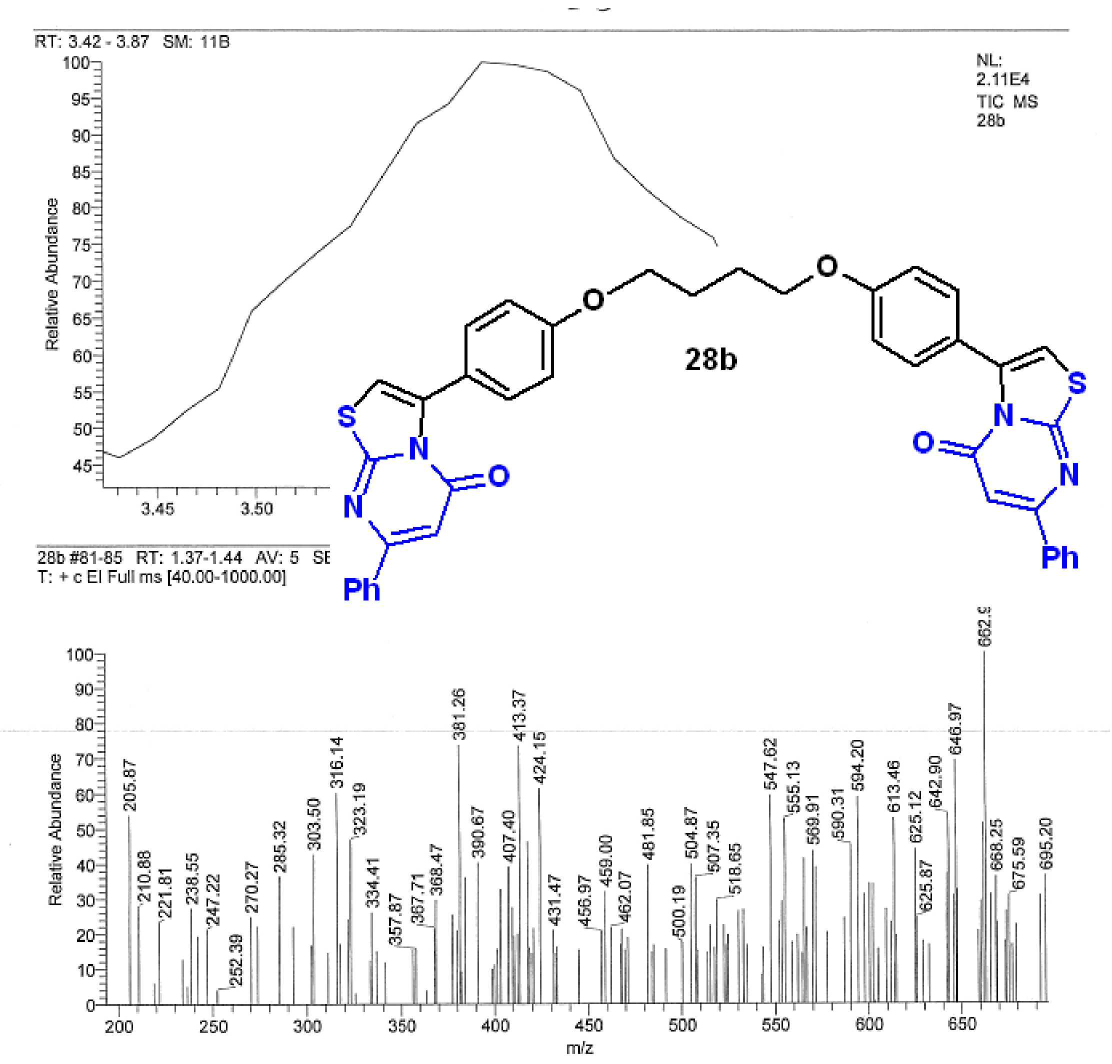

Mass spectrum compound 28b

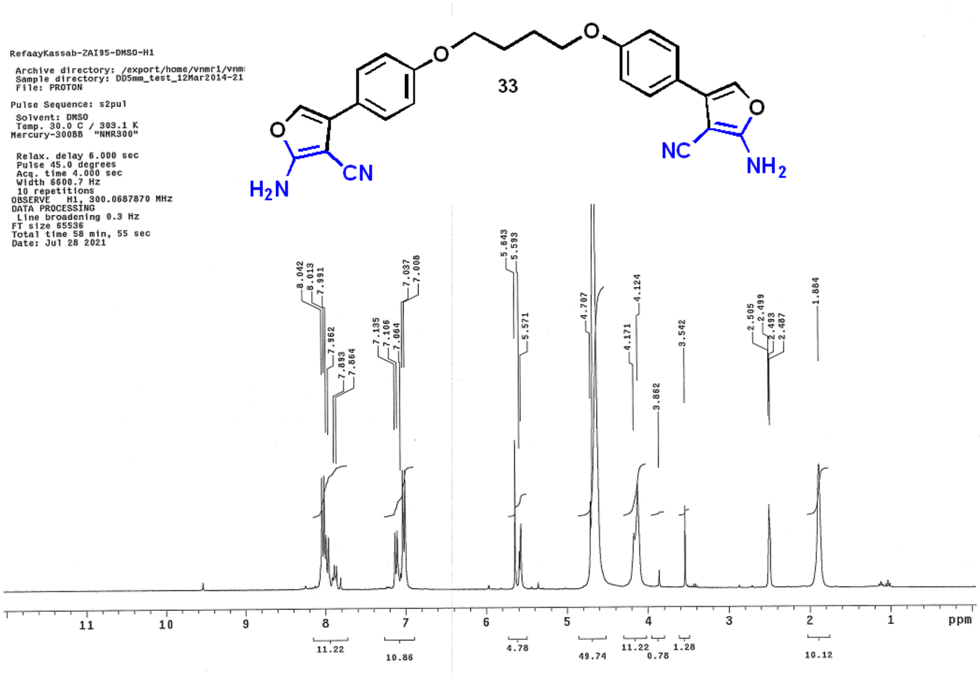

^{1}H NMR spectrum compound 33

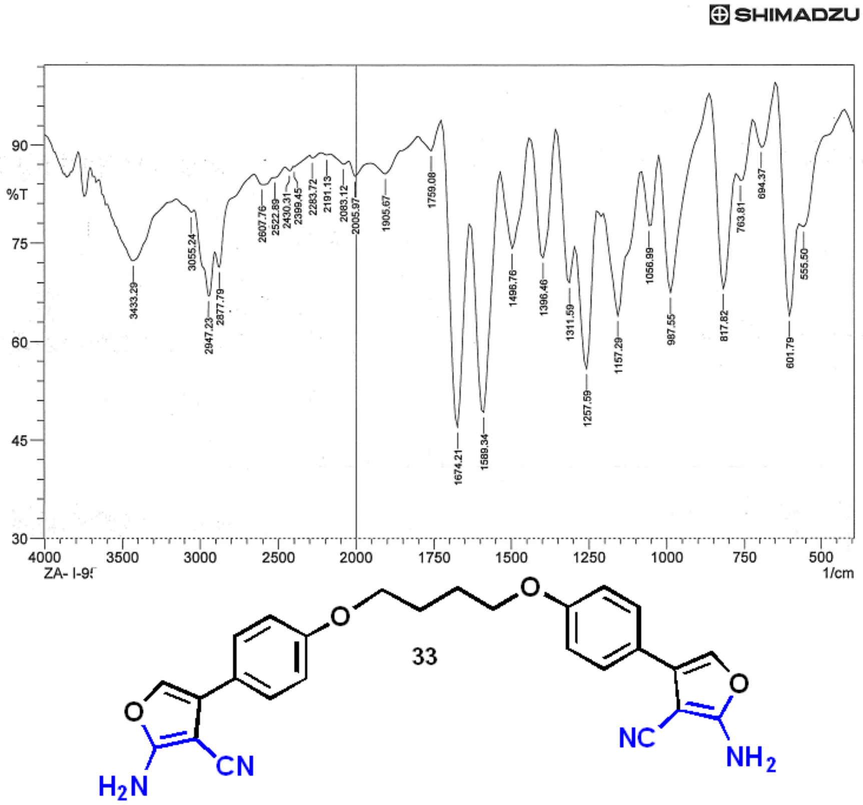

IR spectrum compound 8d

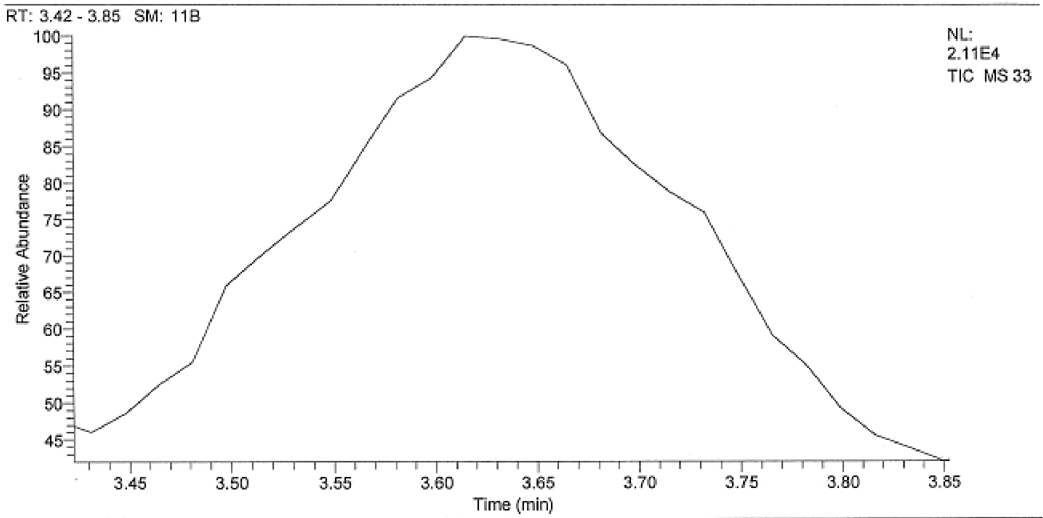

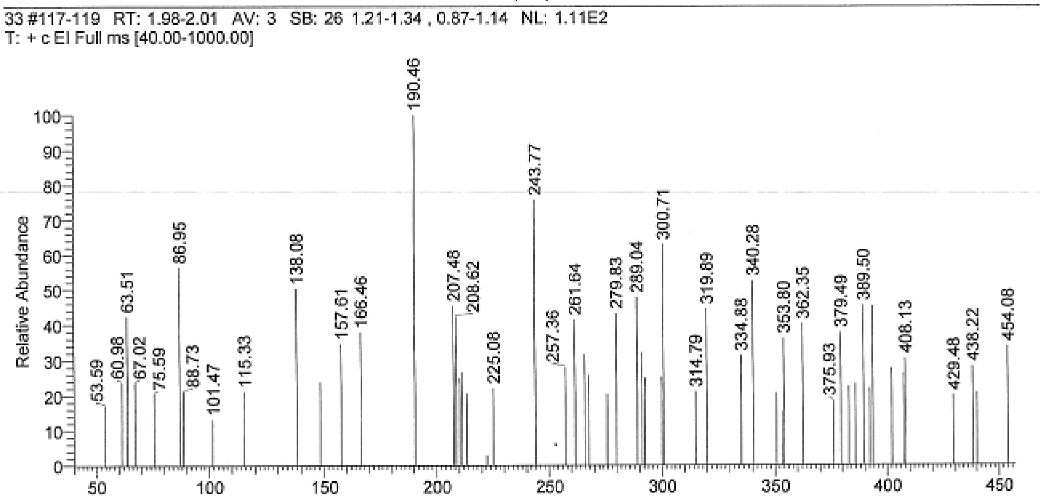

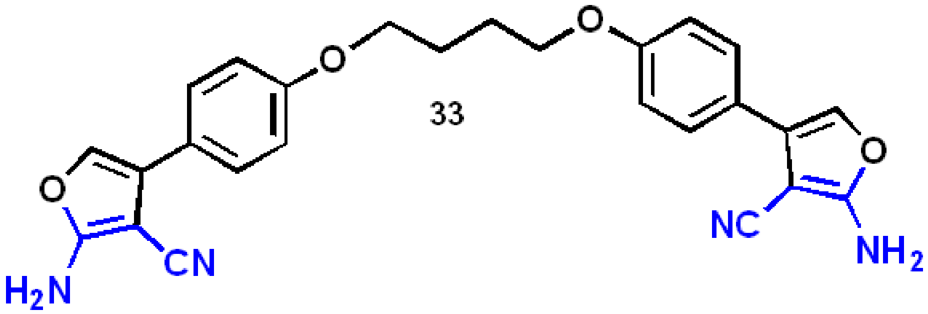

Mass spectrum compound 33

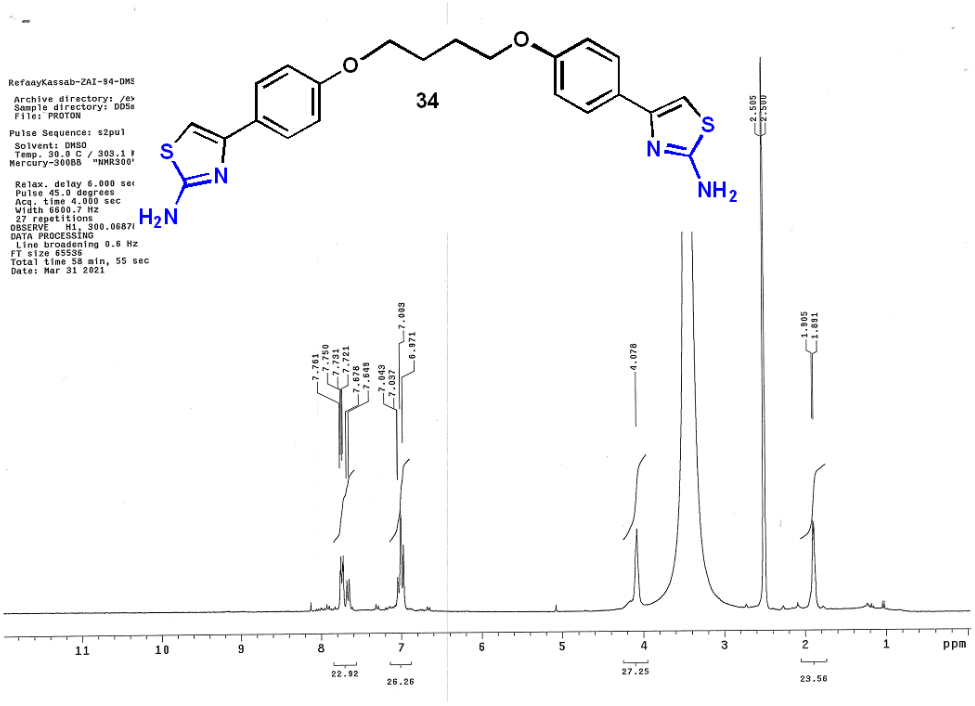

^{1}H NMR spectrum compound 34

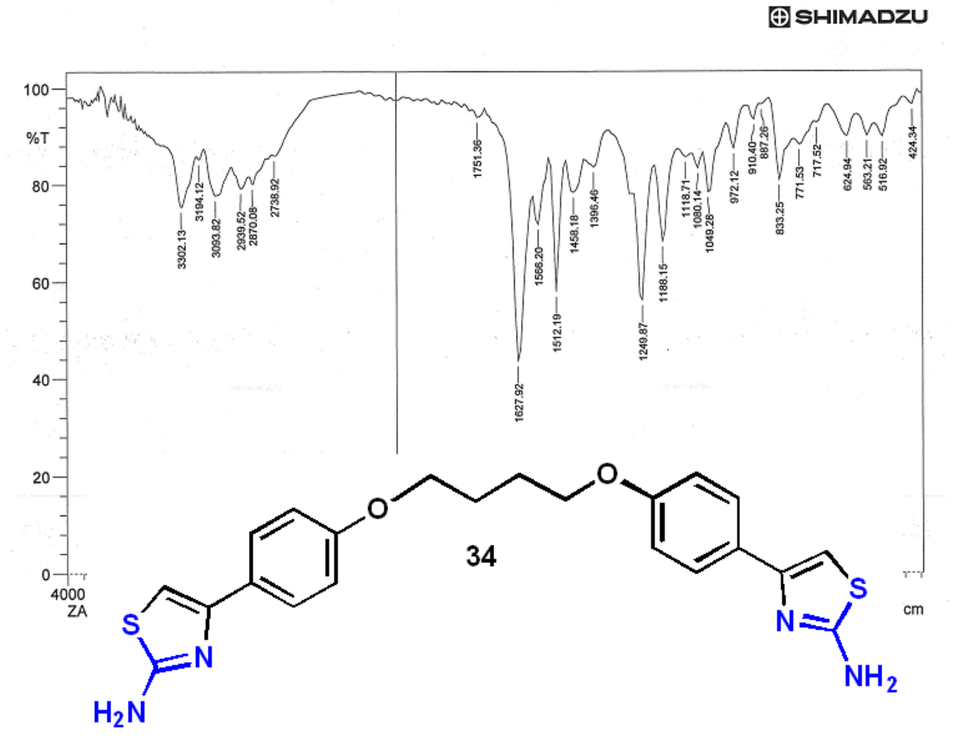

IR spectrum compound 34

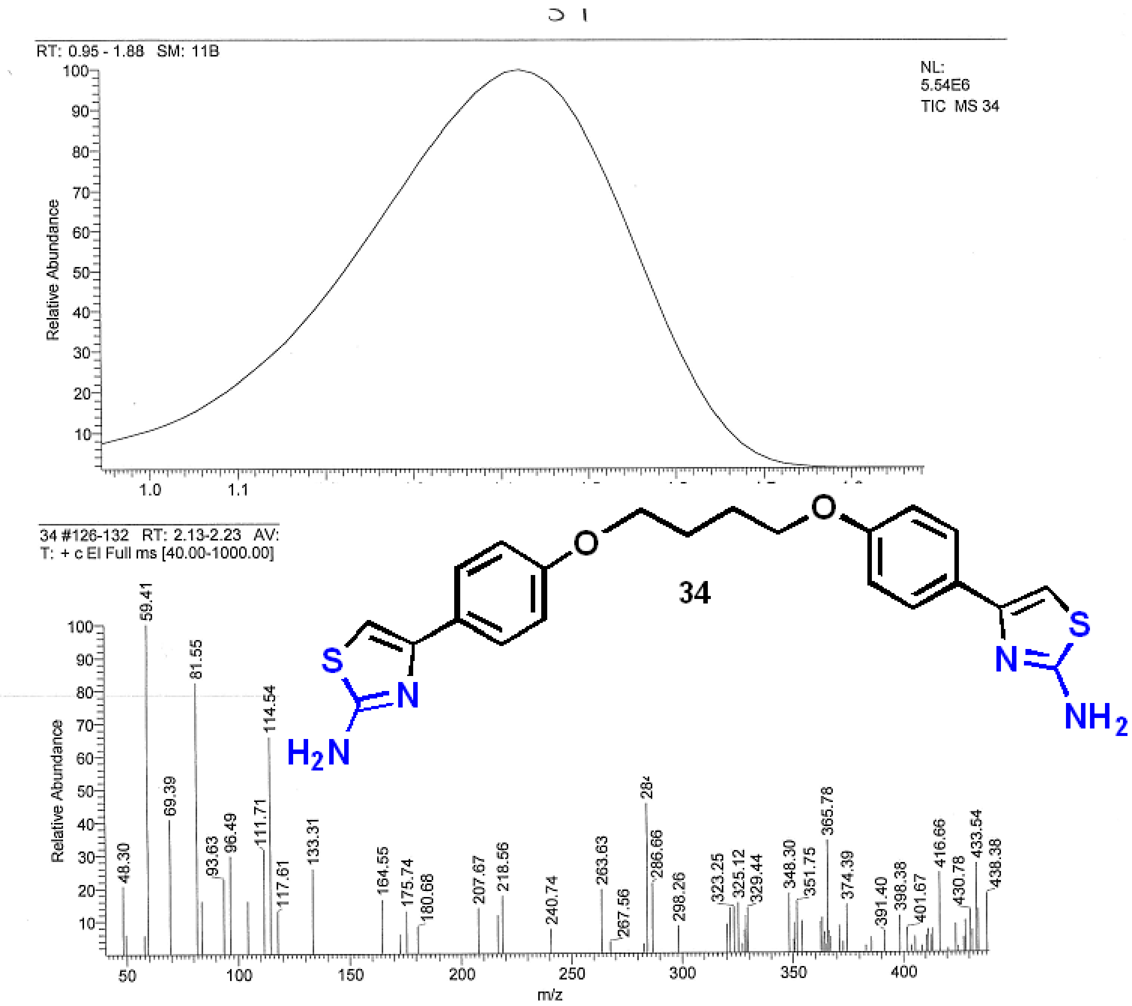

Mass spectrum compound 34

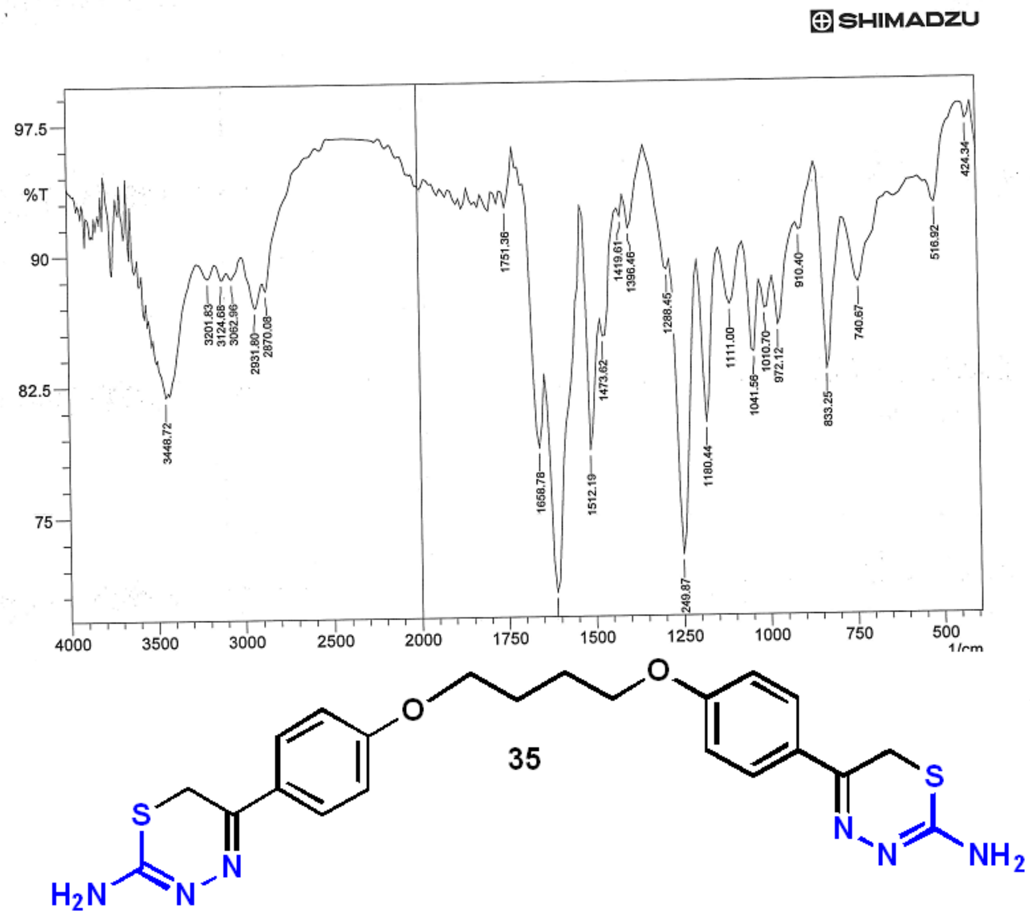

IR spectrum compound 35

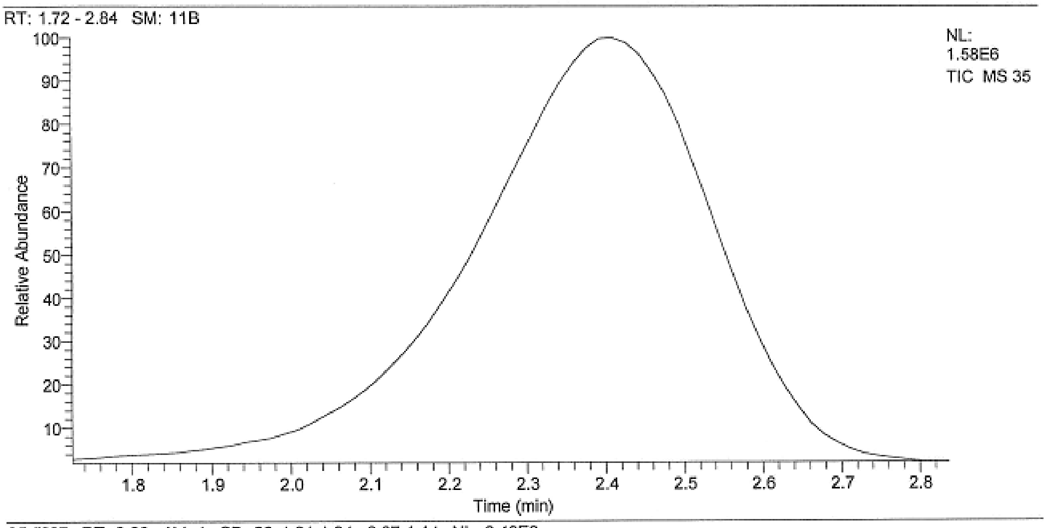

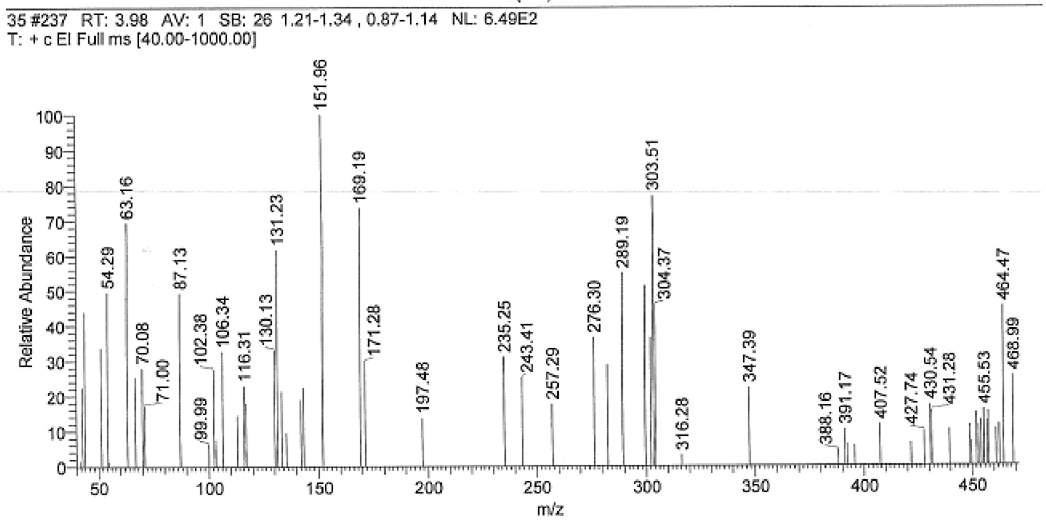

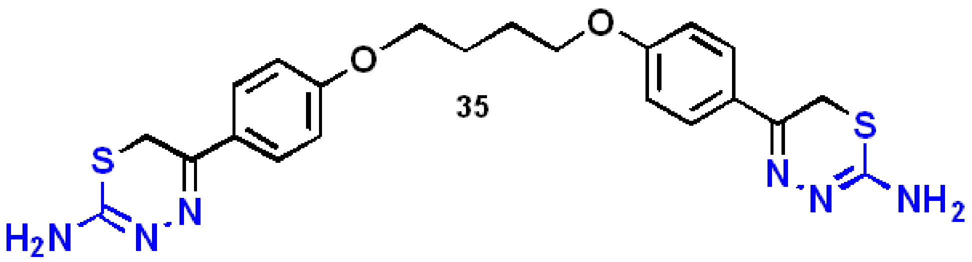

Mass spectrum compound 35

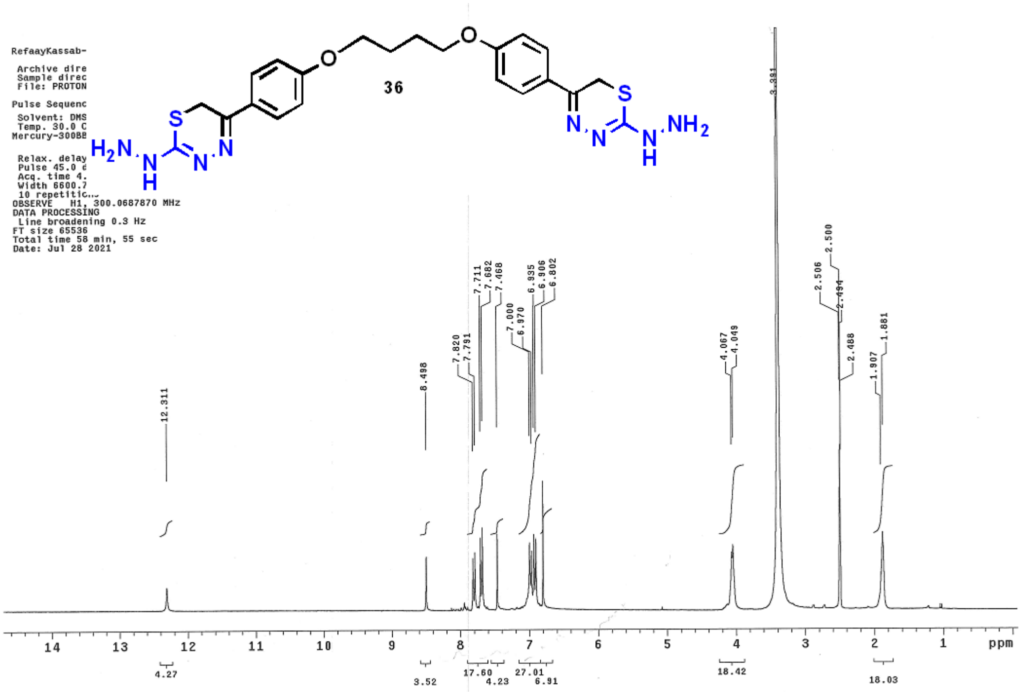

^{1}H NMR spectrum compound 36

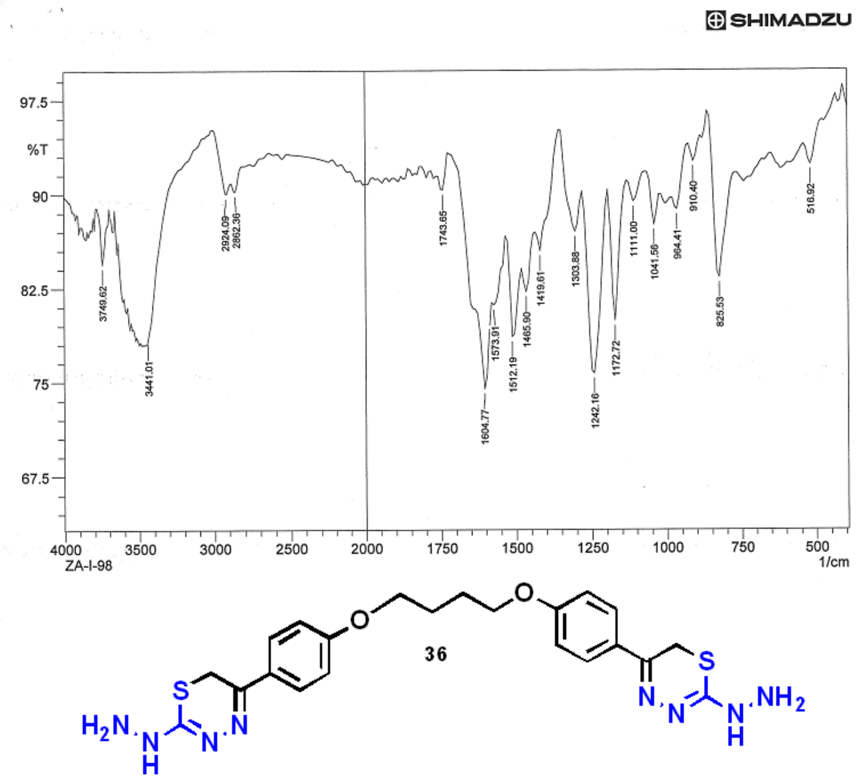

IR spectrum compound 36

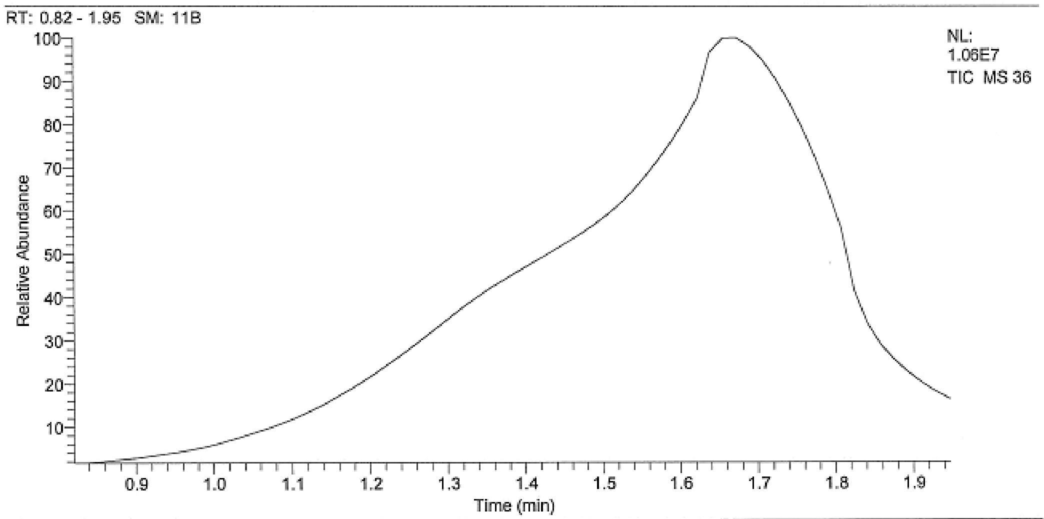

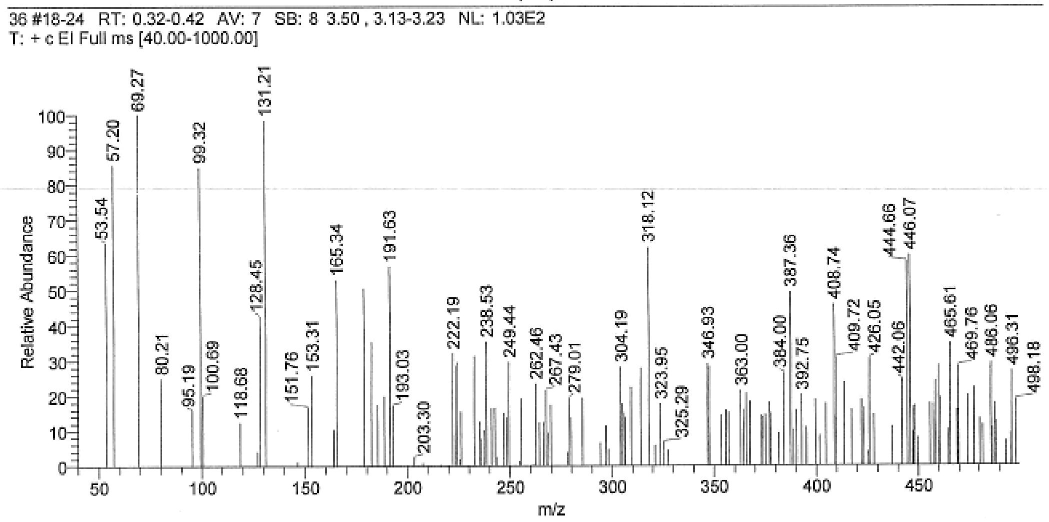

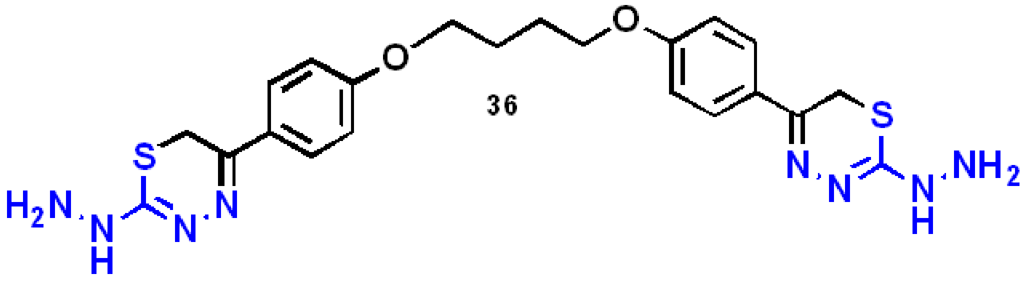

36

Mass spectrum compound 36
